# Role of DARPP-32 and ARPP-21 in the Emergence of Temporal Constraints on Striatal Calcium and Dopamine Integration

**DOI:** 10.1371/journal.pcbi.1005080

**Published:** 2016-09-01

**Authors:** Anu G. Nair, Upinder S. Bhalla, Jeanette Hellgren Kotaleski

**Affiliations:** 1 Science for Life Laboratory, School of Computer Science and Communication, KTH Royal Institute of Technology, Stockholm, Sweden; 2 National Centre for Biological Sciences, Tata Institute of Fundamental Research, Bangalore, India; 3 Manipal University, Manipal, India; 4 Department of Neuroscience, Karolinska Institutet, Solna, Sweden; University of Virginia, UNITED STATES

## Abstract

In reward learning, the integration of NMDA-dependent calcium and dopamine by striatal projection neurons leads to potentiation of corticostriatal synapses through CaMKII/PP1 signaling. In order to elicit the CaMKII/PP1-dependent response, the calcium and dopamine inputs should arrive in temporal proximity and must follow a specific (dopamine after calcium) order. However, little is known about the cellular mechanism which enforces these temporal constraints on the signal integration. In this computational study, we propose that these temporal requirements emerge as a result of the coordinated signaling via two striatal phosphoproteins, DARPP-32 and ARPP-21. Specifically, DARPP-32-mediated signaling could implement an input-interval dependent gating function, via transient PP1 inhibition, thus enforcing the requirement for temporal proximity. Furthermore, ARPP-21 signaling could impose the additional input-order requirement of calcium and dopamine, due to its Ca^2+^/calmodulin sequestering property when dopamine arrives first. This highlights the possible role of phosphoproteins in the temporal aspects of striatal signal transduction.

## Author Summary

A response towards an environmental stimulus could be reinforced if it elicits a reward. On the subcellular level, the environmental stimulus and the reward signal lead to a transient increase in striatal calcium- and dopamine-signaling, respectively. The integration of calcium and dopamine signals, which is important for reward-learning, could elicit a downstream response only if they are close in time and arrive in correct order (first calcium and then dopamine). This study proposes that the requirement for the input signals to be temporally close and in correct order could emerge due to the coordinated signaling via two striatal phosphoproteins, DARPP-32 and ARPP-21. The DARPP-32 signaling implements an input-interval dependent gating function and ARPP-21 implements an input-order dependent threshold-like function. Thus, a molecular mechanism has been presented here which could explain the emergence of important temporal aspects of subcellular signal integration in reward-learning.

## Introduction

Reinforcement learning plays an important role in building the learned-behavioral repertoire of an organism. It operates by updating the salience of an environmental cue or a cue-response association which has elicited a reward in the past. Basal ganglia are critical for reward learning and the input nucleus, striatum, is the locus of integration for the environmental and the reinforcement signals [1]. The environmental stimuli are largely conveyed to the striatum by the cortical glutamatergic afferents which converge onto the striatal medium-sized spiny neurons (MSNs). The incoming glutamatergic activity leads to an influx of calcium ions through N-methyl-D-aspartate receptor (NMDAR) in the postsynaptic MSNs [2–4]. On the other hand, the reinforcement signal is encoded by the dopaminergic inputs from the mid-brain which activates the dopamine D1 receptors (D1R) in one of the MSN populations [5,6].

The postsynaptic integration of NMDAR-mediated calcium and dopamine-dependent D1R signaling leads to the potentiation of corticostriatal synapses on D1R-expressing MSNs, thus resulting in reward-learning [7–9]. Ca^2+^-Calmodulin-dependent kinase II (CaMKII) and Protein-Phosphatase 1 (PP1) signaling plays an important role in this process [9]. There are several substrate proteins, like receptor subunits and translational regulators, which are phosphorylated by CaMKII and dephosphorylated by PP1, and this in turn could influence synaptic strength [10,11]. However, in order to elicit the CaMKII/PP1-dependent downstream response in MSNs, the calcium and dopamine inputs should fulfill two temporal requirements [9]. Specifically, the calcium and dopamine inputs should be in temporal proximity (input-interval constraint) and the dopamine should follow, and not precede, the calcium input (input-order constraint). Thus, only those calcium and dopamine signals which adhere to these constraints are effective to produce corticostriatal potentiation [9]. Despite the physiological significance of these temporal constraints, the molecular mechanism underlying their emergence is not clear.

In this computational study, we investigated the integration of calcium and dopamine signals by D1R-expressing MSNs to explain the emergence of the aforementioned temporal constraints, using quantitative kinetic modeling. Our results suggest that DARPP-32 (**D**opamine and c**A**MP-regulated **P**hospho**p**rotein **32**kDa) could play an important role in the dopamine-dependent gating of the calcium signaling, which aligns with previous experimental observations [9,12]. DARPP-32 is believed to be an important integrator of calcium and dopamine signaling in striatum [13]. According to our simulations, the transient nature of DARPP-32 signaling could be responsible for the emergence of the requirement regarding temporal proximity of calcium and dopamine signals in the integration process. However, it appears that this DARPP-32 mediated signaling alone could not distinguish the temporal order of calcium and dopamine signals. Therefore, it may not be sufficient to explain the emergence of the input-order constraint. We propose that another striatally-enriched phosphoprotein, ARPP-21 (c**A**MP-**R**egulated **P**hospho**p**rotein **21**kDa), has the potential to introduce the input-order dependency into the integration process. ARPP-21, upon dopamine-dependent phosphorylation, has the ability to bind with Ca^2+^/calmodulin, thus affecting the calcium signaling [14,15]. In our signaling model, ARPP-21 imposes the input-order constraint by implementing an input-order dependent threshold-like function for CaMKII activation. Thus, our results predict an important mechanistic role for ARPP-21, whose physiological relevance remains elusive, in the context of striatal reward learning. Moreover, in the case of a multi-trial scenario, an inter-trial refractoriness could also emerge due to ARPP-21 signaling. Thus, this study puts forth a novel, but readily testable, mechanism which could explain various aspects of the striatal calcium-dopamine integration. In general, it also highlights the possible role of regulatory-phosphoproteins in shaping the temporal aspects of subcellular signal integration. Such a phosphoprotein dependent mechanism could represent a more generic signaling motif for different brain regions where DARPP-32 and ARPP-21 are expressed and input timing is crucial.

## Materials and Methods

**Fig 1:**
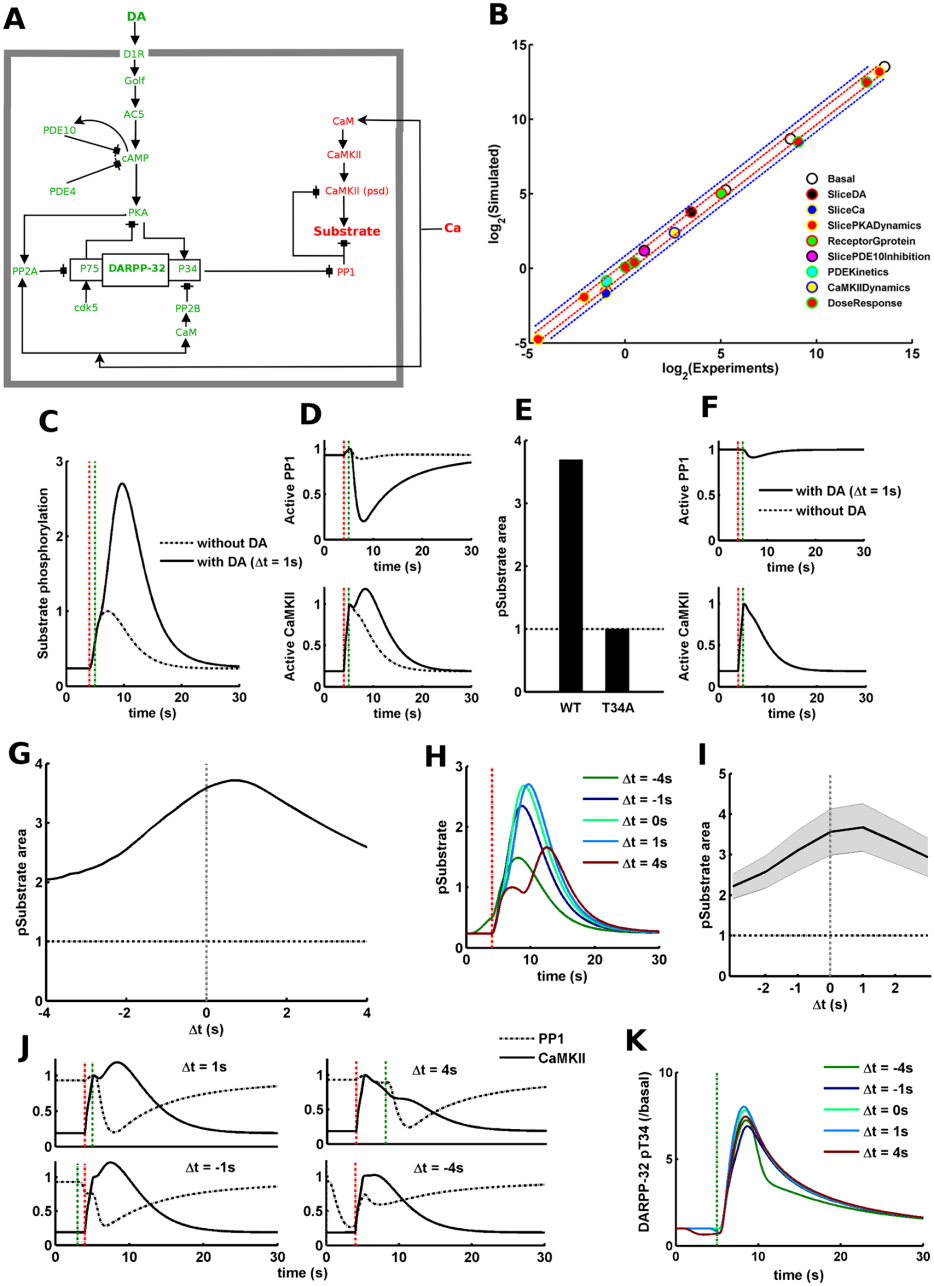
DARPP-32 mediated signaling network shows dopamine-dependent gating of CaMKII/PP1 signaling and input-interval constraint. (A) The standard striatal signaling network modeled using mass-action kinetics. The species in green belong to the dopamine signaling axis whereas the species in red belong to the calcium signaling axis. There is one clear point of cross-talk from the dopamine signaling axis to the calcium signaling axis, P34 (Thr-34) in DARPP-32. (B) Experimental versus simulated values for all phenotypic variables we have used to constrain the signaling model. The legends correspond to the name of the phenotypes as presented in the “Target Molecular Phenotypes” in “Materials and Methods”. Each phenotype contains several plotted points corresponding to the values of different phenotypic variables involved. The experimental and simulated values are plotted as their log_2_ values to keep the plotted points well separated for better visualization. The red and blue dotted lines show the limit for ±30% and ±80% divergence, respectively. (C) The downstream response in terms of the amount of phosphorylated substrate (pSubstrate) for 10 pulses of calcium input at 10 Hz (leading to 5μM calcium concentration) with or without a dopamine (DA) pulse (1.5μM) (1.0s) following the calcium input. Δt = 1s indicates that the start time of dopamine input is 1s after the start time of calcium input. The y-axis is normalized to the amount of pSubstrate produced by calcium alone. The vertical red dotted line marks the start of calcium input. The vertical green dotted line marks the start of dopamine input for the case with DA. (D) Active PP1 and CaMKII traces for calcium input with or without dopamine input. The vertical red and green dotted lines mark the start of calcium and dopamine inputs, respectively. The CaMKII and PP1 levels are normalized with the maximum values observed with calcium input alone, i.e. without dopamine. (E) The normalized area under the curve of pSubstrate response (pSubstrate area) in simulated DARPP-32 wild type (WT) and Thr-34 mutant (T34A) for the case with DA. The y-axis is normalized to the pSubstrate area for calcium input alone. The horizontal dotted line, with y-axis value equals 1, represents the pSubstrate area for calcium input alone. (F) Active PP1 and CaMKII traces in simulated T34A mutant case for calcium input with or without dopamine input. The vertical red and green dotted lines mark the start of calcium and dopamine inputs, respectively. The CaMKII and PP1 levels are normalized with the maximum values observed with calcium input alone, i.e. without dopamine. (G) The normalized area under the curve of pSubstrate response (pSubstrate area) as a function of Δt. Δt is the difference between the start time of dopamine and calcium inputs, (Δt = t_dopamine_−t_calcium_). A positive Δt value means the dopamine input follows the calcium input and vice versa. The y-axis is normalized to the pSubstrate area for calcium input alone. The horizontal dotted line corresponds to the pSubstrate area produced by calcium input alone and the vertical dotted line corresponds to Δt = 0. The amplitude of the calcium and dopamine inputs is 5μM and 1.5 μM, respectively. (H) pSubstrate trace for different Δt. The vertical red dotted line marks the start of calcium input. (I) The normalized mean (solid black line) pSubstrate activation area and associated standard deviation (shaded grey region) produced by two fold increase or decrease in all model parameter values, one at a time. The y-axis is normalized to the pSubstrate area for calcium input alone. The horizontal dotted line corresponds to the pSubstrate area produced by calcium input alone and the vertical dotted line corresponds to Δt = 0. (J) Active CaMKII and PP1 traces for different Δt values. The vertical red and green dotted lines mark the start of calcium and dopamine inputs, respectively. The CaMKII and PP1 levels are normalized with the maximum values observed with calcium input alone, i.e. without dopamine. Each subplot mentions the associated Δt value. The upper panels have the same x-axis values as the lower panels. (K) Timecourse of DARPP-32 phosphorylation for different values of Δt. The vertical green dotted line marks the start of dopamine input. The start time of calcium input depends on the value of Δt relative to the start time of dopamine input. The y-axis is normalized by the basal level of DARPP-32 Thr-34 phosphorylation.

In order to study the integration of calcium and dopamine input signals at MSNs we developed a reaction-kinetic model with cross-talking calcium and dopamine signaling axes, Fig 1A. The basic entities of this subcellular signaling model are individual reactions, which could be either a reversible reaction or an irreversible reaction. Individual reactions are quantitatively modeled as ordinary differential equation (ODE) governed by the mass-action kinetics. For example, the time evolution of chemical species in a typical reversible chemical reaction is formulated as:

$$A+B\;\begin{array}{c} k_{f}\\ ↔\\ k_{r} \end{array}AB$$

$$\frac{-d\left[A\right]}{dt}\;=\;\frac{-d\left[B\right]}{dt}\;=\;\frac{d\left[AB\right]}{dt}\;=\;k_{f}\left[A\right]\left[B\right]-\;k_{r}\left[AB\right]$$

If a reaction is an irreversible reaction then the backward rate constant, k_r_, is set to zero. Thus, the overall signaling model is a collection of such ODEs. The resulting system is solved using the ode15s solver provided by the Simbiology toolbox of MATLAB (MathWorks) with a maximum timestep of 0.01s. This model assumes a well-mixed approximation in a single compartment. Thus, no spatial or molecular heterogeneity has been taken into account.

The topology of the signaling network is built using biochemical reactions which have experimentally known interpretations for the signaling pathways. While it is possible that there are additional reactions and pathways in the actual system, we selected a subset which is consistent with the known striatal literature and pertinent to the currently-studied behavior. In cases where biochemical details were incomplete, we assumed simple mass action reactions that could account for the known molecular dependencies. A detailed description of the signaling network is provided below. After fixing the topology, the free variables left in the system are the reaction kinetic parameters and starting amount for different proteins. The parameters have been constrained using various types of neuronal and, in many cases, MSN-specific data. A list of target phenotypes used to constrain the model is also described below. The model SBML file could be downloaded from BioModels database (Model Id: MODEL1603270000) [16]. Additionally, the list of reactions and the reaction parameters could be found in the supporting file (see Tables A, B and C in S1 Text).

### Modeled Biochemical Network

It is known that the integration of transient calcium and dopamine signals leads to synaptic potentiation of the corticostriatal synapses and this process is mediated by CaMKII activity [8,9,17]. CaMKII activation leads to the phosphorylation of its substrate but this could be counteracted by PP1 [9]. Thus, we read out the phosphorylation level of a generic CaMKII/PP1 substrate to understand the calcium-dopamine integration.

#### Calcium signaling

The sequence of calcium-triggered signaling events modeled in this study is shown in Fig 1A (red font). A calcium input leads to the activation of calmodulin into Ca^2+^/calmodulin. The transition of calmodulin to Ca^2+^/calmodulin is modeled as a sequential two-step reaction. Each step results in the binding of calcium to one of the calcium-binding domains in calmodulin. The parameters for each of the steps are factored assuming a double binding-site Adair-Klotz’s model fitted to the known calcium calmodulin binding data [18]. At each step of the calcium-calmodulin binding, the partially or fully calcium-bound calmodulin could bind to its target. Thus, the reactions for calcium, calmodulin and target forms two cyclic reaction sets and the parameters for these cyclic reactions have been constrained to fulfill the thermodynamic requirement (or detailed balance) as previously described [19].

In our model, an important Ca^2+^/calmodulin target is CaMKII. Under the basal condition, the inactive CaMKII is known to be enriched in the F-actin rich cytoplasmic compartment [20]. On the other hand, the activation of CaMKII reduces its affinity to F-actin thereby resulting in its translocation to the near-synaptic/ postsynaptic-density (PSD) compartment [21]. In our signaling model, we represent CaMKII in these two different compartments using two separate pools of CaMKII. A first order reversible reaction between these CaMKII pools is used to model the translocation of respective forms of CaMKII between the F-actin rich compartment and the PSD. The active CaMKII has a higher abundance in the PSD compartment whereas the inactive form mostly resides in the F-actin rich compartment [20]. These observations have been considered to estimate the parameters for the translocation-reaction.

The activation of CaMKII leads to its autophosphorylation thereby further increasing its affinity to Ca^2+^/calmodulin [22]. The autophosphorylation of the CaMKII increases its affinity to the PSD compartment where it could be dephosphorylated by PP1 [23]. We modeled the autophosphorylation of CaMKII according to a previously described method [24]. Additionally, this autophosphorylation is believed to be important for the cooperative activation of CaMKII by Ca^2+^/calmodulin [25], which indeed is the case in our signaling model. Recently, it has also been shown that the CaMKII activation in response to a transient calcium input is short-lived and deactivates with a predominantly fast time constant (few seconds) [9,26,27]. The CaMKII activation parameters in the current model have also been constrained to take into account this transient nature of CaMKII activation with a deactivation time constant of (~6s) [26].

The active CaMKII leads to the phosphorylation of its substrates and many of these substrates are dephosphorylated by PP1 [10,11]. To capture this we used a generic CaMKII/PP1 substrate in the model. Since we are interested in the effect of CaMKII on the synaptic proteins, the generic CaMKII/PP1 substrate in the modeled signaling network is assumed to be localized in the near-synaptic/PSD compartment. Thus, this substrate could only be phosphorylated by the active CaMKII which has been translocated to the PSD compartment.

#### Dopamine signaling

The sequence of dopamine-triggered signaling events modeled in this study is shown in Fig 1A (green font). This dopamine D1-dependent signaling module is an updated version of a previous model [28], recalibrated to accommodate additional experimental observations. In this signaling network, dopamine leads to the activation of D1 receptors which in turn activates G_olf_ G-proteins [29,30]. The activated G_olf_ further predominantly activates Adenylyl Cyclase type V (AC5) which leads to an increased level of cAMP [29,31]. The activation of the receptors and G-proteins appears to be significantly faster in neurons compared to other non-neuronal cells [32–37]. Thus, the receptor and G-protein parameters in the current signaling model have been optimized to take into account the fast kinetics.

Under the basal condition the cAMP levels are expected to be low in the D1R expressing MSNs due to the low basal receptor activity. The basal cAMP level estimated for different types of neurons with low AC activity is around 30–90 nM [38,39]. In striatum, the cAMP elevation has been observed to be very sensitive to transient D1R activation [40]. Moreover, it has also been recently observed that a steady state activation of the striatal D1 receptors lead to a very high concentration of cAMP (~10μM) [41]. We have considered these quantitative data to estimate the reaction parameters related to basal and G_olf_-dependent AC5 activation. The parameters for the cAMP degradation by phosphodiesterases (PDEs) have also been refined in the current version of the model to match the known PDE kinetics observed in live cells [42]. It is also known that PDE10 is an important PDE in MSNs and the effective inhibitory strength of this PDE is constrained in the model by MSN specific data [43]. The elevation in cAMP level further leads to the activation of PKA signaling [44]. Similar to cAMP, the striatal PKA signaling appears to be sensitive to transient D1R activation [40]. Furthermore, the activation/deactivation kinetics of the PKA signaling also appears to be significantly faster in MSNs [9,40]. These MSN-specific PKA data have been used to further refine the PKA kinetic-parameters.

PKA activation in MSNs leads to the phosphorylation of DARPP-32 at its Thr-34 residue and this turns DARPP-32 into a potent inhibitor of protein phosphatase PP1 [45]. DARPP-32 could also be phosphorylated at Thr-75 by cdk5 which turns it into an inhibitor of PKA [46]. The basal level of Thr-34 phosphorylated DARPP-32 is around 0.4μM and Thr-75 phosphorylation is around 12μM in striatum [46–48]. However, upon the application of saturating amount of D1R specific agonist the level of Thr-34 increases by 11X whereas the Thr-75 level decreases by 0.5X [48–50]. It has also been observed that the level of both Thr-34 and Thr-75 decrease upon the application of saturating amount of NMDA [51,52]. Similar to the previous versions of the cAMP/PKA/DARPP-32/PP1 signaling module, this version is also constrained by the above mentioned basal and stimulated level of various DARPP-32 states. The kinetic parameters of the DARPP-32 signaling have been further sped up for the current version of the model in the light of recent experimental observations [9]. These recently published experiments suggest that signaling via DARPP-32 could rapidly affect the downstream target, within a few seconds (~4-5s) [9].

#### Addition of ARPP-21 into the dopamine signaling axis

**Fig 2:**
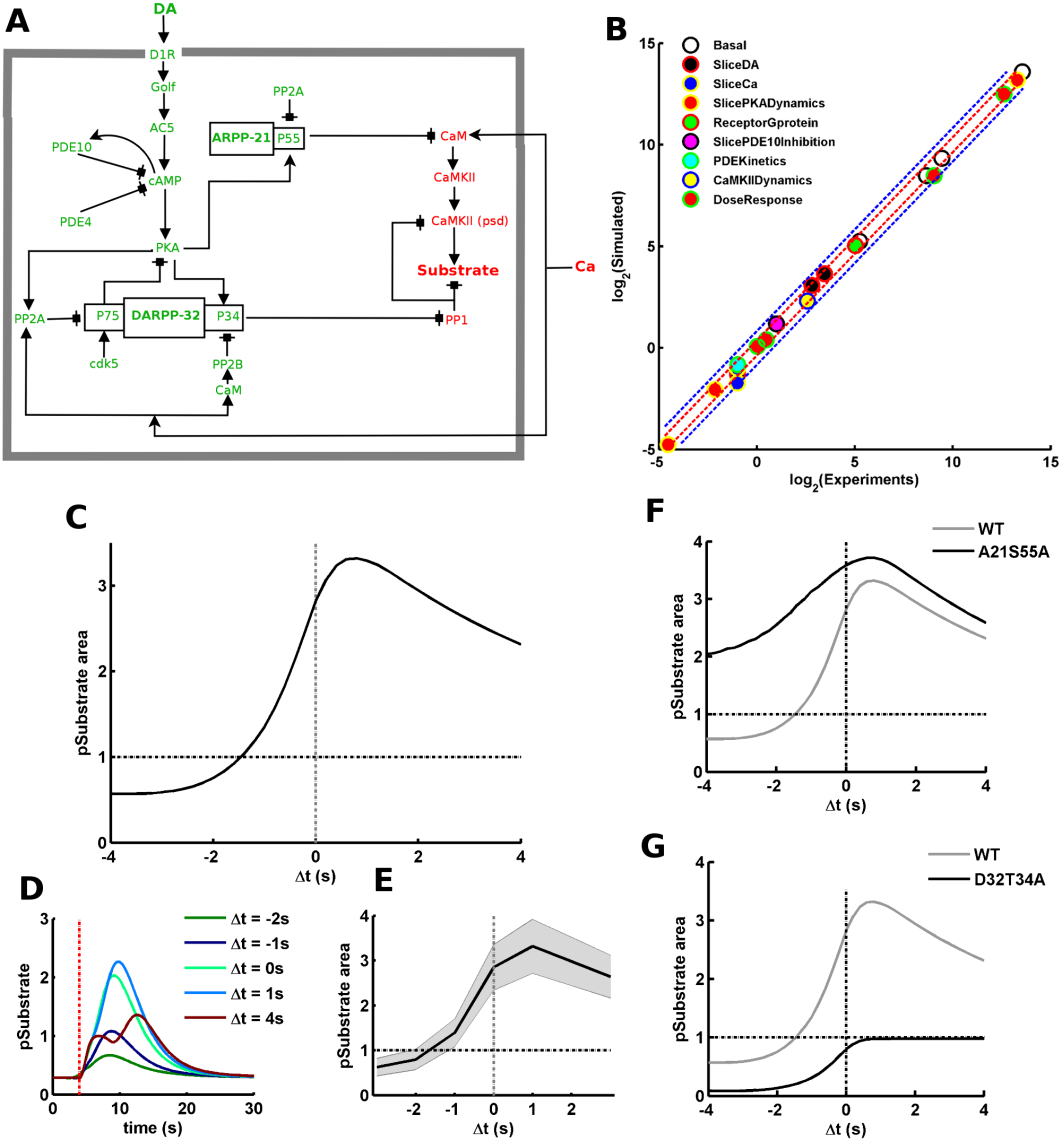
Addition of ARPP-21 into the signaling network confers input-order constraint. (A) The updated signaling model with the addition of ARPP-21. The species in green belong to the dopamine signaling axis whereas the species in red belong to the calcium signaling axis. There are two clear points of cross-talk from the dopamine signaling axis to the calcium signaling axis, one is P34 (Thr-34) in DARPP-32 and the second is P55 (Ser-55) in ARPP-21. (B) Experimental versus simulated values for all phenotypic variables. Experimental versus simulated values for all phenotypic variables we have used to constrain the signaling model (including the ARPP-21 data). The legends correspond to the name of the phenotypes as presented in the “Target Molecular Phenotypes” in “Materials and Methods”. Each phenotype contains several points corresponding to different phenotypic variables. The experimental and simulated values are plotted as their log_2_ values to keep the plotted points well separated for better visualization. The red and blue dotted lines show the limit for ±30% and ±80% divergence, respectively. (C) Strong temporal ordering is seen in the normalized area under the curve of pSubstrate response as a function of Δt. The y-axis is normalized to the pSubstrate area for calcium input alone. Δt specifies the interval between the start of calcium and dopamine inputs. A negative interval suggests that dopamine precedes calcium whereas a positive interval suggests that dopamine follows calcium. The horizontal dotted line corresponds to the pSubstrate area produced by calcium input alone and the vertical dotted line corresponds to Δt = 0. The amplitude of the calcium and dopamine inputs is 5μM and 1.5 μM, respectively. (D) pSubstrate trace for different Δt. The vertical red dotted line marks the start of calcium input. (E) The mean (solid black line) of normalized pSubstrate activation area and associated standard deviation (shaded grey region) produced by two fold increase or decrease in individual parameter values, one at a time. The y-axis is normalized to the pSubstrate area for calcium input alone. The horizontal dotted line corresponds to the pSubstrate area produced by calcium input alone and the vertical dotted line corresponds to Δt = 0. (F) The normalized area under the curve of pSubstrate response as a function of Δt for simulated wildtype (WT) and ARPP-21 mutant (A21S55A). The y-axis is normalized to the pSubstrate area for calcium input alone. The horizontal dotted line corresponds to the pSubstrate area produced by calcium input alone and the vertical dotted line corresponds to Δt = 0. (G) The normalized area under the curve of pSubstrate response as a function of Δt for wildtype (WT) and DARPP-32 mutant (D32T34A). The y-axis is normalized to the pSubstrate area for calcium input alone. The horizontal line corresponds to the pSubstrate area produced by calcium input alone and the vertical line corresponds to Δt = 0.

As described in the “Results” sections, our results indicate that the DARPP-32-mediated dopamine signaling alone is insufficient to explain the emergence of certain temporal constraints of the calcium-dopamine integration (results corresponding to Fig 1G). Thus, we considered some additions to the existing core of the cAMP/PKA/DARPP-32 signaling. We included ARPP-21 and its regulation into the signaling network, Fig 2A.

ARPP-21 is a striatally enriched phosphoprotein similar to DARPP-32 [53]. The estimated amount of total ARPP-21 in striatal homogenate is around 17–20 μM [15,53]. We assume the total amount of ARPP-21 to be 20 μM in our signaling model. ARPP-21 is phosphorylated at its Ser-55 residue by the D1R-dependent cAMP/PKA and dephosphorylated by PP2A [54,55]. The phosphorylated ARPP-21 could then bind to Ca^2+^/calmodulin, thus acting as a competitive inhibitor for other Ca^2+^/calmodulin dependent proteins [15]. Even though the binding affinity between ARPP-21 and Ca^2+^/calmodulin is not known in cellular context, it is clearly high enough to reduce the activation of other cellular Ca^2+^/calmodulin targets [15]. A comparison between the changes in striatal phosphorylated and dephosphorylated form of ARPP-21 upon stimulation with saturating level of forskolin suggests that the basal level of phosphorylated ARPP-21 is around 700nM [14,54]. D1R specific agonist application leads to a 4X increase in the phosphorylated form of ARPP-21 in striatal homogenate [54]. However, this does not give an MSN specific fold change of ARPP-21 phosphorylation. Assuming that the basal phosphorylated levels to be low in both MSN types and the D1 type MSN is responsible for the D1R agonist dependent increase in the phosphorylation, the D1 MSN specific stimulated phosphorylation is around 7X. We estimated this using a previously discussed neuron specific correction for striatal MSNs [50]. We used all these quantitative and qualitative information to constrain the ARPP-21 related reaction-parameters.

### Target Molecular Phenotypes

Several quantitative molecular phenotypes have been used to constrain the model. Similar to the previous modeling studies, here also the term “phenotype” has been used to refer a set of observables in a specific experimental setting [28,50]. These target phenotypes, most of which are MSN specific, have been collected from published literature and are listed in Table 1. The phenotypes which we used are either one-time measurement for a specific stimulus (“SliceDA”, “SliceCa”, “SlicePDE10Inhibition”; refer Table 1), time-series measurement (“ReceptorGProtein”, “SlicePKADynamics”, “PDEKinetics”, “CaMKIIDynamics”; refer Table 1) or dose response (“DoseResponse”; refer Table 1). The comparison between the model output and the experimental data for the one-time measurements has been done using fold change of the respective marker. The time-series data have been compared between the model and experiments by parameterizing them into simple functions, like monoexponentials (“ReceptorGProtein”, “PDEKinetics”, “CaMKIIDynamics”) and difference of two exponentials (“SlicePKADynamics”). The dose response data has been parameterized with Hill equation for the purpose of comparison between the model and experiment. Apart from comparing the stimulated state of various effectors, the model has also been constrained by the known basal level of various effectors (“Basal”; refer Table 1). The total amounts of various species have also been taken from published literature wherever possible. In the case of the model without ARPP-21 (Fig 1A), the ARPP-21 related phenotypic variables (in “Basal” and “SliceDA”) have been ignored. Figs 1B and 2B show the comparison between the experimental and simulated values of phenotypic variables for the signaling models without and with ARPP-21. The differences are minor.

**Table 1:** Molecular phenotypes used to constrain the model.

Molecular Phenotypes				
Phenotype Name	Treatment	Marker	Phenotypic Variables and Values	References
**Basal**	No specific treatment. The basal state of the system.	cAMP	30–90 nM	[38,39]
		DARPP-32p34	~400 nM	[47,48]
		DARPP-32p75	~12000 nM	[46,48]
		ARPP-21p55	~700 nM	[14,54]
**ReceptorGProtein**	Saturating concentration of ligand.	G-protein activation (activation time constant between 10–100 ms)	Monoexponential fit: k = 10–100 s^-1^	[32,34]
**SliceDA**	Striatal slice + dopamine (≥ 10μM). Sampled at 5 min.	DARPP-32p34	11 X basal	[48,49]
		DARPP-32p75	0.5 X basal	
		ARPP-21p55	7 X basal	[54]
**SliceCa**	Striatal slice + NMDA (100μM). Sampled at 10 min.	DARPP-32p34	0.5 X basal	[51,52]
		DARPP-32p75	0.5 X basal	
**SlicePKADynamics**	Striatal slice + high D1R agonist (equivalent to dopamine ≥ 10μM)	cAMP	~10000 nM	[41]
	Striatal slice + transient dopamine signal (VTA dopamine neuron stimulation).	AKAR (PKA/PP1 substrate)	Difference of two exponentials: k1 = 0.043s^-1^; k2 = 0.227s^-1^	[9]
**SlicePDE10Inhibition**	Striatal slice + PDE10 inhibitor (papaverine 10μM)	DARPP-32p34	2 X basal	[43]
**PDEKinetics**	Saturating concentration of cAMP (50μM) + PDEs in the system.	cAMP	Monoexponential fit: k = 0.508s^-1^	[42]
**CaMKIIDynamics**	Cultured neurons + transient calcium input.	CaMKII deactivation	Monoexponential fit: k = 0.166s^-1^ (τ = 6s)	[26]
**DoseResponse**	Protein + varying concentration of ligand.	PKA Activation (cAMP as ligand)	Hill’s fit: h = 1.4; K = 527.8 nM	[44]
		Ca•Calmodulin (Ca as ligand)	Hill’s fit: h = 1; K = 6290 nM	[18]

### Inputs to the Signaling Network

The current biochemical-reaction model considers two input signals: (1) dopamine and (2) calcium. The dopamine input represents increased extracellular dopamine concentration due to the burst activity of dopaminergic neurons in response to an unexpected reward [5]. Such a burst could lead to a dopamine peak of around 1.5μM amplitude [56]. We use the same amplitude in our model. The basal concentration of dopamine is assumed to be 20nM in this study. On the other hand, the calcium input in the model represents an increase in the intracellular calcium concentration in response to 10 glutamate-triggered calcium pulses at 10 Hz. These calcium input parameters are in accordance with the previously published experiments where the temporal requirement of calcium and dopamine integration have been observed [9]. The raise and decay time-constants of individual calcium pulses are obtained from the observed kinetics of intracellular NMDAR calcium [57]. The amplitude is set such that the maximum intracellular calcium concentration reaches around 5μM. The basal concentration of the intracellular calcium is assumed to be 60nM in this study.

## Results

As described above, the integration of striatal calcium and dopamine inputs leads to the phosphorylation of downstream CaMKII/PP1 substrates in MSNs. This, however, requires that the inputs should fulfill certain temporal requirements [9]. Specifically, the calcium and dopamine inputs should arrive close in time (input-interval constraint), and the dopamine input should follow calcium (input-order constraint). DARPP-32 is known to be important in this process [9]. Thus, we first examined the mechanistic role of DARPP-32 in the CaMKII/PP1 signaling. DARPP-32 could explain the emergence of the input-interval but not significantly the input-order constraint. We then incorporated ARPP-21 into the signaling network and this led to the emergence of the input-order constraint. Next, we explored the mechanism through which ARPP-21 implements this input-order constraint. Finally, we investigated implications and predictions of the proposed signaling mechanisms.

### DARPP-32 Signaling Promotes the CaMKII/PP1 Substrate Phosphorylation

To investigate the integration of dopamine and calcium inputs by the subcellular signaling processes in the D1R expressing MSNs, we developed a kinetic model of the standard striatal signaling network, Fig 1A [28,50,58–60]. This kinetic model is comprised of a dopamine/D1R/AC5/cAMP/PKA/DARPP-32 and a calcium/calmodulin/CaMKII signaling axes (refer “Materials and Methods”), and it has been constrained to closely match various known experimental observations, Fig 1B (refer “Target Molecular Phenotypes” in “Materials and Methods”). In order to understand the downstream effect of calcium-dopamine integration on the CaMKII/PP1 signaling, we read out the phosphorylation level of a generic CaMKII/PP1 substrate while simulating the signaling network. This substrate is phosphorylated by CaMKII and dephosphorylated by PP1, Fig 1A.

We first looked at how dopamine could affect the calcium triggered CaMKII signaling. To this end, we measured the difference between the CaMKII/PP1 substrate phosphorylation produced by a transient calcium input (elevation in intracellular calcium concentration; refer “Materials and Methods”) with and without an accompanying transient dopamine input (elevation in dopamine concentration; refer “Materials and Methods”). The dopamine input, in this case, follows the calcium input after 1s.

The simulation results indicate that the level of substrate phosphorylation is significantly higher when the calcium transient is paired with a dopamine transient compared to the response produced by the calcium alone, Fig 1C. This suggests that a transient dopamine input could gate the calcium triggered CaMKII/PP1-dependent signaling. Since the substrate phosphorylation depends on the coordinated activity of CaMKII and PP1, we looked at how the input signals affect the level of active CaMKII and PP1 to get further insights. The simulations suggest that calcium leads to CaMKII activation in both the cases with and without dopamine, though there are slight differences, Fig 1D (lower panel). On the other hand, the level of active PP1, unlike CaMKII, shows a larger difference between the two cases, Fig 1D (upper panel). The level of active PP1 remains high throughout the simulation without much difference when the model is presented only with the calcium input, Fig 1D. However, the active level of PP1 is transiently reduced when a dopamine input is also presented to the model along with the calcium input, Fig 1D.

Under the basal condition, the substrate is under dephosphorylation pressure due to the high PP1 level. This is in line with recent experimental observations [9]. Our simulations suggest that even though a calcium input alone could lead to CaMKII activation thereby increasing the phosphorylation of the substrate, this is not sufficient enough to counteract the dephosphorylation pressure of PP1. The inhibition of PP1, produced by a concomitant dopamine input, could transiently relieve the dephosphorylation pressure which in turn leads to a significant increase in the resulting substrate phosphorylation and a slight increase in CaMKII activation (Fig 1C and 1D).

Dopamine dependent PKA activation leads to the phosphorylation of Thr-34 residue of DARPP-32 and this turns DARPP-32 into a potent inhibitor of PP1 [61]. Thus, to verify whether DARPP-32 is responsible for the dopamine-dependent increase in substrate phosphorylation, we simulated the knocked down of the Thr-34 phosphorylation of DARPP-32 (T34A) in our model by removing the respective reaction. A comparison of the substrate response between the default (wildtype; WT) and the mutant (T34A) models indeed suggests that the dopamine dependent gating is mediated by the phosphorylation of Thr-34, Fig 1E. In the T34A mutant case, there is no difference between the substrate response produced by calcium alone and calcium accompanied by a dopamine input, Fig 1E. As expected, there is no difference in either active CaMKII or PP1 between with and without dopamine in the case of T34A mutant, Fig 1F.

### DARPP-32 Mediated Signaling Could Implement the Input-Interval Constraint

Using the aforementioned signaling network, we then investigated how the substrate phosphorylation depends on the temporal relation between calcium and dopamine inputs. The interval between the inputs is referred as Δt, such that Δt = (t_dopamine_—t_calcium_), where t_calcium_ and t_dopamine_ are the start time of calcium and dopamine transients, respectively. A small absolute value of Δt indicates that the calcium and dopamine inputs are close in time. A positive value of Δt means that the calcium input is followed by the dopamine input and vice-versa. This relation between the substrate response and Δt could shed light on whether the input-interval and input-order constraints exist in this signaling network. If the substrate response is higher for smaller absolute values of Δt compared to large Δt values then this suggests the existence of an input-interval constraint. In other words, calcium and dopamine should be close in time for effective downstream activation. Similarly, if substrate response is higher for positive values of Δt compared to negative values then this indicates the existence of input-order constraint, i.e. the calcium input should be followed by the dopamine input for effective downstream response.

Our simulations with different Δt values suggest that there appears to be a relation between the substrate phosphorylation and Δt, Fig 1G. For smaller absolute values of Δt the substrate phosphorylation is generally higher, Fig 1G and 1H. As the absolute value of Δt increases the downstream response declines, Fig 1G. Thus, these results suggest that the temporal proximity between the calcium and dopamine inputs is important for an effective dopamine-dependent gating thereby indicating the existence of an input-interval constraint in this signaling network. However, there is a slight asymmetry in the gating function around Δt = 0, e.g. the amplitude of the phosphorylated substrate for Δt = 1s is slightly higher that the amplitude at Δt = -1s, Fig 1H. For longer values of positive Δt (e.g. Δt = 4s), the timecourse of substrate phosphorylation appears to be biphasic, Fig 1H. The first phase corresponds to the start of phosphorylation process under the dephosphorylation pressure of PP1 and the second phase represents the facilitation of the substrate phosphorylation due to the dopamine dependent inhibition of PP1.

Since the signaling network built in this study is quite detailed and contains several parameters, we looked at the robustness of our result towards uncertainty in the parameter-values. In order to test the robustness of the relation between Δt and downstream substrate phosphorylation, we made a 2-fold increase or decrease in the individual parameter value, one at a time, and then simulated the perturbed model. The distribution of the results arising from parameter perturbations suggests that the Δt-dependence in this signaling network is quite robust, Fig 1I.

We then investigated the reason for the emergence of the relation between Δt and downstream substrate phosphorylation. Since the effective substrate phosphorylation depends on CaMKII and PP1, we looked at the behavior of CaMKII and PP1 in order to identify the reason for the existence of the input-interval constraint, Fig 1J. Similar to the above result (Fig 1D), there is CaMKII activation and DARPP-32-mediated PP1 inhibition in response to calcium and dopamine inputs, respectively, Fig 1J (Δt = 1s). This CaMKII activation and PP1 inhibition could be seen for different values of Δt (1s, 4s, -1s, -4s), Fig 1J. However, the coincidence between the time-window of CaMKII activation and PP1 inhibition is considerably different for various Δt, Fig 1J. This overlap between the CaMKII activation and the PP1 inhibition decreases with an increase in the interval between the calcium and dopamine inputs thus reducing the downstream effect, Fig 1J (Δt = 4s, -4s). This difference in the temporal overlap between the CaMKII and PP1 response leads to the Δt-dependence of the substrate phosphorylation response.

As mentioned previously, the PP1 inhibition is produced by dopamine dependent DARPP-32 phosphorylation at Thr-34. DARPP-32 is also dephosphorylated by PP2B in response to calcium. Thus, it may be possible that different Δt values may lead to different level of DARPP-32 phosphorylation thereby affecting the PP1 inhibition. Thus, we also looked at how the level of phosphorylated DARPP-32 changes for different values of Δt. The simulations indicate that DARPP-32 phosphorylation is not very sensitive to various Δt values around zero, Fig 1K. For negative values of Δt, there is a visible calcium dependent DARPP-32 dephosphorylation. However, this effect is quite small relative to the overall response. Thus, the Δt-dependence of the downstream substrate response in our simulations is largely produced by the difference in the temporal overlap between the transient CaMKII and DARPP-32 mediated PP1 response rather than any regulation of the relative strength of DARPP-32 phosphorylation.

The response in this DARPP-32 mediated signaling network depends mostly on the magnitude rather than the sign of Δt, i.e. there is a substantial amount of downstream response even for negative values of Δt, Fig 1G. Thus, the DARPP-32 signaling makes no significant distinction between whether dopamine input precedes or follows the calcium input as long as they are temporally close. In other words, this signaling network could account for the input-interval constraint but not the input-order constraint of the striatal calcium-dopamine integration.

### Addition of ARPP-21 to the Signaling Network Could Produce the Input-Order Constraint

Since the DARPP-32-containing signaling network was not sufficient to explain the input-order constraint, we considered an important addition to it. Apart from DARPP-32, the striatal MSNs also express significant amounts of ARPP-21 [55]. As mentioned above, ARPP-21 has the potential to act as a point of cross-talk between the calcium and dopamine signaling. It could compete with Ca^2+^/calmodulin dependent proteins for the shared calmodulin resource pool [15] upon dopamine dependent phosphorylation [54]. Thus, we included ARPP-21 into the signaling network to test whether it has the ability to introduce the input-order constraint, Fig 2A (refer “Materials and Methods”). This updated model has also been constrained to closely match various known experimental observations including the known striatal ARPP-21 data, Fig 2B (refer “Target Molecular Phenotypes” in “Materials and Methods”).

We then used this updated model to explore the relation between Δt and the substrate phosphorylation. Fig 2C shows the relation between Δt and the substrate phosphorylation for this updated signaling model. The addition of ARPP-21 significantly altered the relation between Δt and the substrate phosphorylation compared to the “without ARPP-21” network (compare Fig 2C with Fig 1G). For the updated signaling network, positive and preferably smaller values of Δt produced higher substrate phosphorylation, Fig 2C. Fig 2D shows the traces of substrate phosphorylation produced for different Δt. Unlike the previous case, the downstream response is highly sensitive to the sign of Δt. If dopamine comes before calcium (Δt < −0.5s) then the downstream response is significantly lower that the corresponding positive Δt response, Fig 2C. Thus, the addition of ARPP-21 confers the calcium-dopamine integration process with the previously unexplained input-order constraint. We assessed the robustness of the updated signaling network by changing the parameter values, one at a time, by 2-folds. The distribution of the results arising from parameter perturbations suggests that the Δt-dependence in this updated signaling network is also quite robust, Fig 2E. The simulation of Ser-55 mutant of ARPP-21 (A21S55A) shows that the phosphorylation of this residue is specifically important for the emergence of the input-order constraint, Fig 2F.

In the previous section, we presented that DARPP-32 mediated signaling promotes the CaMKII/PP1 signaling and explain the input-interval constraint on the calcium-dopamine integration. In the current section, we illustrated that including ARPP-21 into the network could lead to the appearance of the input-order constraint in addition to the input-interval requirement. When taken together, the emergence of both input-interval and input-order constraints could be explained by the coordinated activity of DARPP-32 and ARPP-21. The role of DARPP-32 and ARPP-21 could be further clarified by simulating the signaling network with loss-of-function mutation in DARPP-32 and ARPP-21. A comparison between simulated wild-type (WT) and Ser-55 mutant of ARPP-21 (A21S55A) shows that loss-of-function mutation in ARPP-21 abolishes the input-order interval by significantly affecting the response in the negative Δt region, Fig 2F. On the other hand, the difference between the response of WT and Thr-34 mutated DARPP-32 (D32T34A) suggests that DARPP-32 is responsible for the overall dopamine dependent gating of the CaMKII/PP1 dependent substrate phosphorylation, Fig 2G. There is no significant substrate response in the case of DARPP-32 mutation, Fig 2G.

### ARPP-21 Acts by Implementing an Input-Order Dependent Threshold-Like Function for CaMKII Activation

As shown above, the addition of ARPP-21 into the signaling network could introduce the input-order constraint by significantly reducing the downstream response for the negative Δt regime, Fig 2C and 2D (compare with Fig 1G). Here, we illustrated the mechanism with which the updated model with ARPP-21 enforces this input-order constraint.

**Fig 3:**
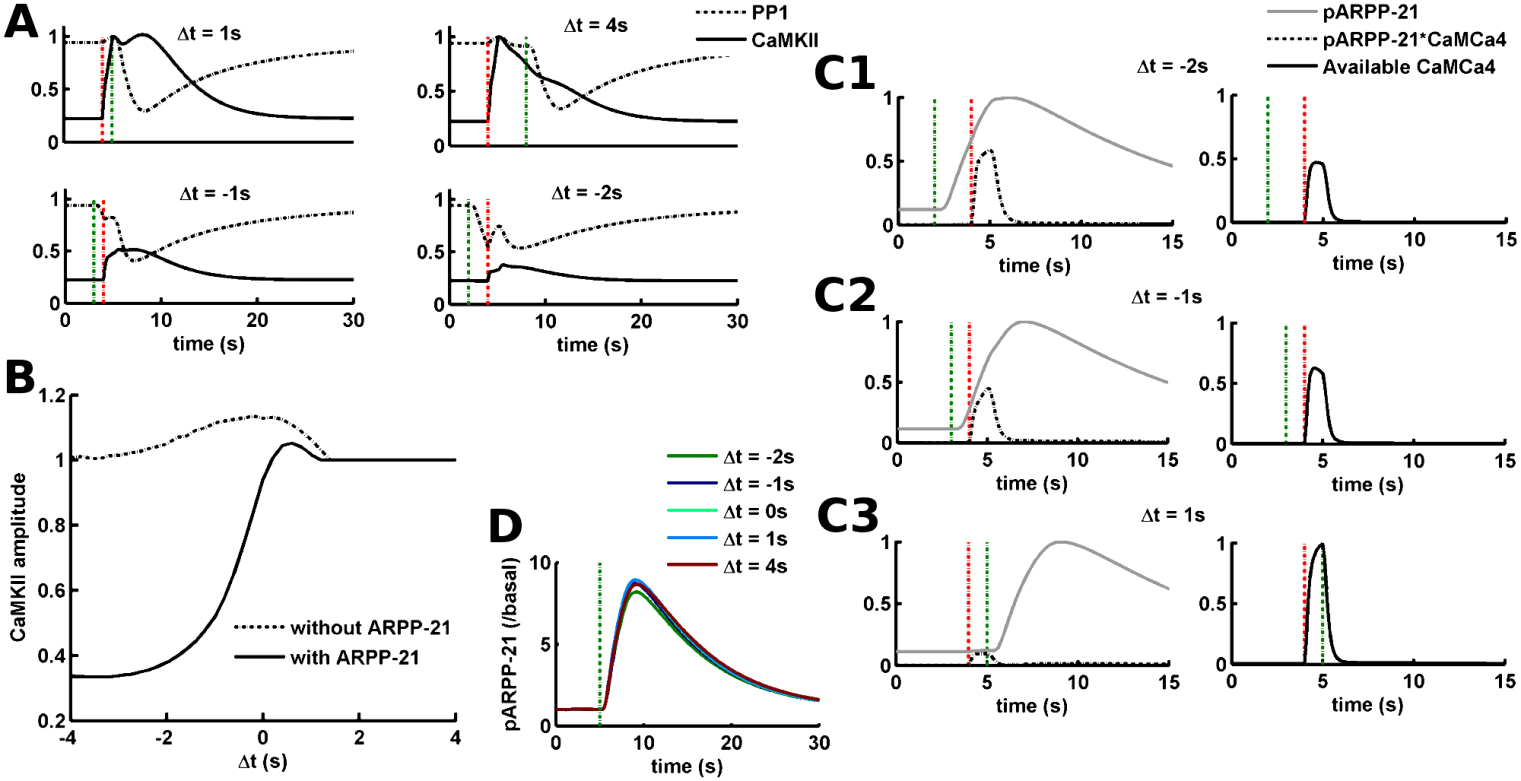
Mechanism for the emergence of input-order constraint. (A) Active CaMKII and PP1 traces for different Δt values. The vertical red and green dotted lines mark the start of calcium and dopamine inputs, respectively. The CaMKII and PP1 levels are normalized by the maximum values observed with calcium input alone, i.e. without any dopamine input. Each panel mentions the associated Δt value. The upper panels have the same x-axis values as the lower panels. (B) The amplitude of active CaMKII as a function of Δt with or without ARPP-21 in the modeled system. The y-axis is normalized by the amplitude of CaMKII produced by the calcium input alone. (C) Traces for ARPP-21 phosphorylation (pARPP-21), pARPP-21 sequestration of Ca^2+^/calmodulin (pARPP-21*CaMCa4) and Ca^2+^/calmodulin available for CaMKII activation (Available CaMCa4) for three different Δt, (C1) Δt = -2s, (C2) Δt = -1s and (C3) Δt = 1s. The left figure panels show pARPP-21 and pARPP-21*CaMCa4 whereas the right panels show available CaMCa4. The vertical red and green dotted lines mark the start of calcium and dopamine inputs, respectively. pARPP-21 and pARPP-21*CaMCa4 are normalized to the maximum level of total phosphorylated ARPP-21. The available CaMCa4 is normalized by the maximum available CaMCa4 with calcium input alone. (D) Timecourse of ARPP-21 phosphorylation for different values of Δt. The vertical green dotted line marks the start of dopamine input. The start time of calcium input depends on the value of Δt relative to the start time of dopamine input. The y-axis is normalized by the basal level of phospho-ARPP-21.

We looked at how active CaMKII and PP1 levels are affected for different Δt values to understand the relation between substrate phosphorylation and Δt for this updated signaling network, Fig 3A. The CaMKII and PP1 activity patterns for positive Δt values (Δt = 1s and Δt = 4s) are not significantly different between the updated network (Fig 3A (upper panel)) and the network without ARPP-21 (Fig 1J (upper panel)). However, there is a significant reduction in the CaMKII activation for negative Δt values in the updated signaling network, Fig 3A (lower panel; compare with Fig 1J (lower panel)). There appears to be an input-order dependent threshold-like relation between the amplitude of active CaMKII and Δt, Fig 3B. Specifically, low CaMKII activation level for negative Δt and a sudden increase as the Δt becomes positive. This sigmoid-dependence of CaMKII activation on Δt is mediated by ARPP-21 as shown by the difference between with and without ARPP-21 case, Fig 3B.

We then investigated how the ARPP-21 could reduce the CaMKII activation in the negative Δt regime, i.e. only when dopamine input precedes the calcium input. It is known that dopamine leads to the phosphorylation of Ser-55 residue on ARPP-21 and this phosphorylation turns ARPP-21 into an excellent binding partner of Ca^2+^/calmodulin [15]. This could suggest a possibility of Ca^2+^/calmodulin sequestration by ARPP-21 thus reducing the CaMKII activation. To ascertain this possibility we first considered the case of Δt = -2s as an example, Fig 3C1. In this case, the dopamine input precedes the calcium input by 2s. The dopamine input leads to the phosphorylation of ARPP-21 and by the time the calcium input arrives there is already a significant level of phosphorylated ARPP-21 (pARPP-21), Fig 3C1 (left panel). As expected the calcium transient leads to an increase in the level of Ca^2+^/calmodulin. A significant part of the Ca^2+^/calmodulin binds to the pARPP-21 produced by the preceding dopamine, Fig 3C1 (left panel). This effectively reduces the amount of Ca^2+^/calmodulin available for CaMKII activation, Fig 3C1 (right panel). This reduction in the available Ca^2+^/calmodulin leads to a lower CaMKII activation. Thus, the sequestration of Ca^2+^/calmodulin by pARPP-21, thereby reducing its availability, indeed explains the low CaMKII activation in the negative Δt regime.

As the interval between dopamine and calcium decreases in the negative Δt region, e.g. Δt = -1s, the sequestration of Ca^2+^/calmodulin by pARPP-21 is also reduced, Fig 3C2 (left panel). This in turn leads to more Ca^2+^/calmodulin available for CaMKII activation, Fig 3C2 (right panel). This is due to the lower level of pARPP-21 at the time of the calcium input; compare between Δt = -2s and Δt = -1s, Fig 3C1 and 3C2. On the other hand, the situation is quite different for a positive Δt value, Δt = 1s. In this case, there is no significant level of pARPP-21 at the time of calcium incidence because the dopamine input has not yet arrived to phosphorylate ARPP-21, Fig 3C3 (left panel). Thus, the sequestration of Ca^2+^/calmodulin by ARPP-21 is negligible, Fig 3C3 (left panel), and the Ca^2+^/calmodulin available for CaMKII activation is not reduced, Fig 3C3 (right panel). Therefore, the CaMKII activation is affected by the phosphorylation of ARPP-21 only if the value of Δt is negative, i.e. when dopamine input precedes the calcium input. The level of ARPP-21 phosphorylation itself does not appear to be dependent on the value of Δt, Fig 3D. The level of phosphorylated ARPP-21 starts to increase 1s after the arrival of dopamine input and reaching its maximum in ~4s. Thus, the downstream CaMKII activation due to any calcium input arriving after the dopamine input with a delay of around 1s or greater, i.e. Δt < = -1, is significantly dampened.

### The Phosphorylation of ARPP-21 Predicts an Inter-trial Refractory Period

As highlighted in the previous section, the dopamine-dependent phosphorylation of ARPP-21 results in the dampening of a subsequent calcium-triggered CaMKII activation. In a two-trial scenario, this could mean that the ARPP-21 which is phosphorylated in the first trial may affect the CaMKII activation in the second trial. To test this cross-talk between successive trials we considered the calcium-dopamine integration in a two-trial scenario. The two trials are separated in time by an inter-trial interval (ITI). Each of the trials is represented by a pair of calcium and dopamine inputs with Δt = 1s.

**Fig 4:**
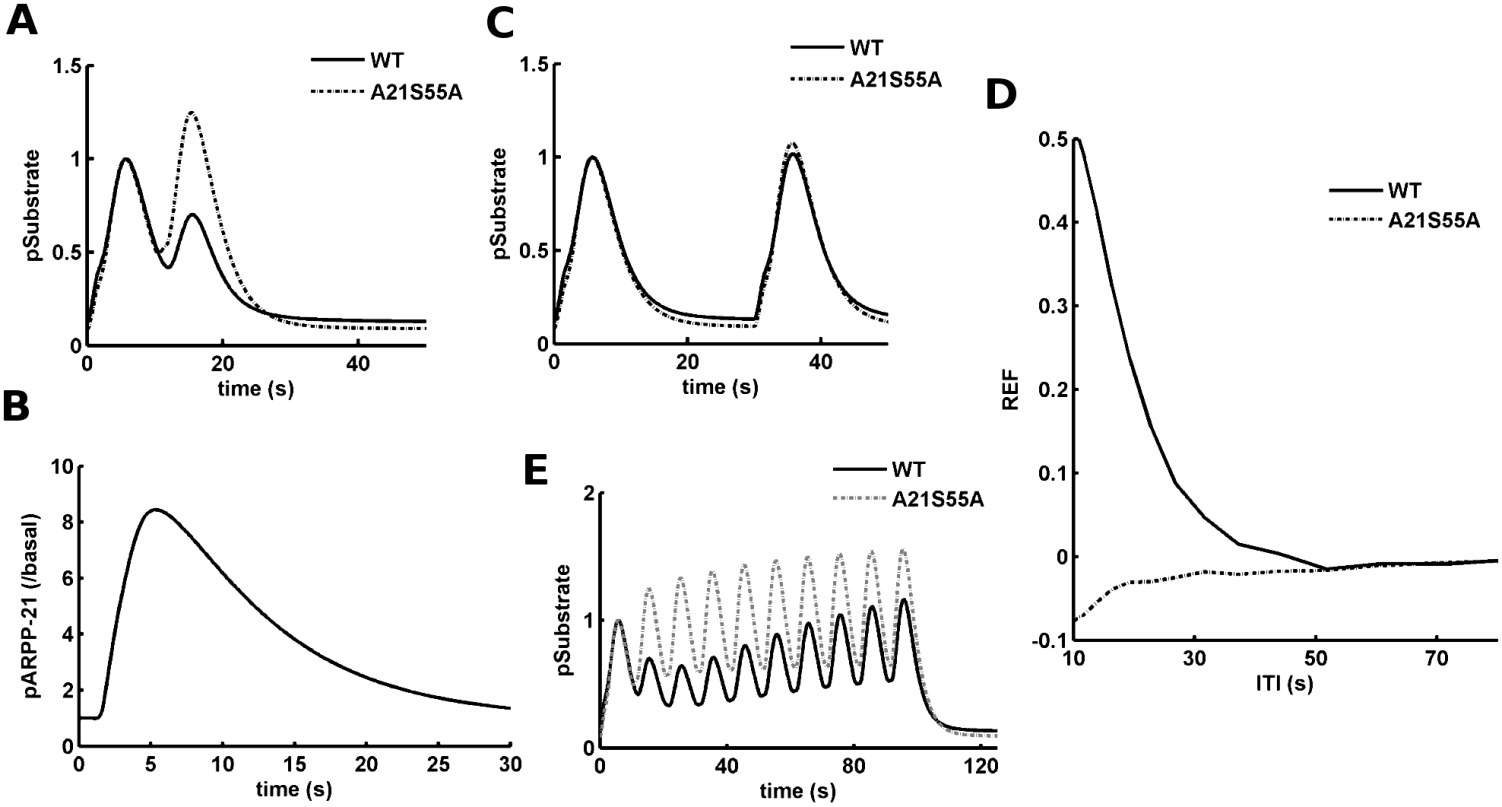
Inter-trial inhibitory effect of ARPP-21 in a two-trial scenario. (A) The downstream response in terms of the amount of phosphorylated substrate (pSubstrate) for the two trial scenario with an inter-trial interval of 10s. The input to each trial consists of calcium and dopamine inputs with Δt = 1s. The y-axis is normalized to the amount of pSubstrate produced in the first trial. The solid line represents the simulated wild type (WT) and the dashed line represents the ARPP-21 mutant (A21S55A). (B) The ARPP-21 phosphorylation (pARPP-21) produced by the first trial. The y-axis is normalized by the basal level of pARPP-21. (C) The downstream response in terms of the amount of phosphorylated substrate (pSubstrate) for the two trial scenario with an inter-trial interval of 30s. The input to each trial consists of 10 pulses of calcium input at 10 Hz followed by dopamine with Δt = 1s. The y-axis is normalized to the amount of pSubstrate produced in the first trial. The solid line represents the wild type (WT) and the dashed line represents the ARPP-21 mutant (A21S55A). (D) Refractoriness (REF) as a function of inter-trial interval (ITI). The solid line represents the wild type (WT) and the dotted line represents the ARPP-21 mutant (A21S55A). (E) The downstream response in terms of the amount of phosphorylated substrate (pSubstrate) for a 10 trial scenario with an inter-trial interval of 10s. The input to each trial consists of calcium and dopamine input with Δt = 1s. The y-axis is normalized to the amount of pSubstrate produced in the first trial. The solid line represents the simulated wild type (WT) and the grey dashed line represents the ARPP-21 mutant (A21S55A).

We first tested a scenario with an ITI = 10s. In this scenario, the substrate response produced by the first trial was similar to the response observed in a single trial case (as in the previous sections), Fig 4A (first peak), but the response produced by the second trial was significantly reduced, Fig 4A (second peak). The response reduction in the second trial was abolished in the case of the simulated ARPP-21 Ser-55 mutation (A21S55A), Fig 4A. This suggests that ARPP-21 phosphorylation could indeed affect the response produced in a subsequent trial. Fig 4B shows the phosphorylation of ARPP-21 in response to a single dopamine input response. There is a transient elevation in the level of ARPP-21 and then it returns to the basal level in 30s. Thus, phosphorylation dependent inhibitory effect of ARPP-21 on the second trial may not be present if ITI is longer that 30s. To test this we simulated the two trial scenario with an ITI = 30s. As expected, there was no significant difference between the responses of the two trials in this case, Fig 4C. Thus, it appears that having ARPP-21 in the signaling network could impose an inter-trial refractoriness for the Ca^2+^/calmodulin-dependent downstream response in an ITI dependent fashion.

We define a metric called refractoriness (REF) to quantify the ITI dependent effect of the first trial on the response of the second trial. REF is defined as follows:

$$REF\left(t\right)\;=\;1-\;\frac{{\left(Activation\;Area\;in\;second\;trial\right)}_{ITI\;=\;t}}{{\left(Activation\;Area\;in\;second\;trial\right)}_{ITI\;=\;100}}$$

where *(Activated Area in second trial)*_*ITI = t*_ is the area under the curve for substrate phosphorylation in the second trial which is separated by the first trial with an ITI = *t* seconds. *(Activated Area in second trial)*_*ITI = 100*_ is the activation area for second trial with an ITI = 100s. The trials with ITI = 100s are considered as well separated trials because with this ITI the second trial response is not affected by the first trial. Thus, if REF = 1, then the response in the second trial is fully eliminated due to the inhibitory effect of the first trial and if REF = 0, then there is no effect of the first trial on the response of the second trial. On the other hand, a negative value for REF indicates a potentiating effect of the first trial on the second trial.

The simulations suggest that the REF decreases as a function of ITI, i.e. REF is higher for lower ITI values and it decreases as ITI increases Fig 4D. Thus, for some range of ITI the two trails do not act independent of each other but as the ITI increases each trial becomes more decoupled. Simulation of the ARPP-21 mutant, A21S55A, suggests that the relation between REF and ITI in this model appears due to the phosphorylation of ARPP-21, Fig 4D.

It could be possible that in a more complex scenario (e.g. 10 trials in a row) the downstream response may integrate over the trials thereby overcoming the refractoriness imposed by pARPP-21. To test this possibility, we implemented a 10 trial scenario with ITI = 10s. Similar to the two trial scenario, each trial consists of 10 calcium pulses at 10 Hz followed by a dopamine input with Δt = 1s. The simulation for this 10 trial scenario suggests that there appears to be a small build of substrate response over the trials, Fig 4E. However, this integration over the trials is relatively small. A comparison between the wild-type and A21S55A scenario suggests that a strong ARPP-21 dependent refractoriness exists in this massed scenario, as well.

### The Input-Interval and Input-Order Constraints Are Robust to the Strength of Input Signals

**Fig 5:**
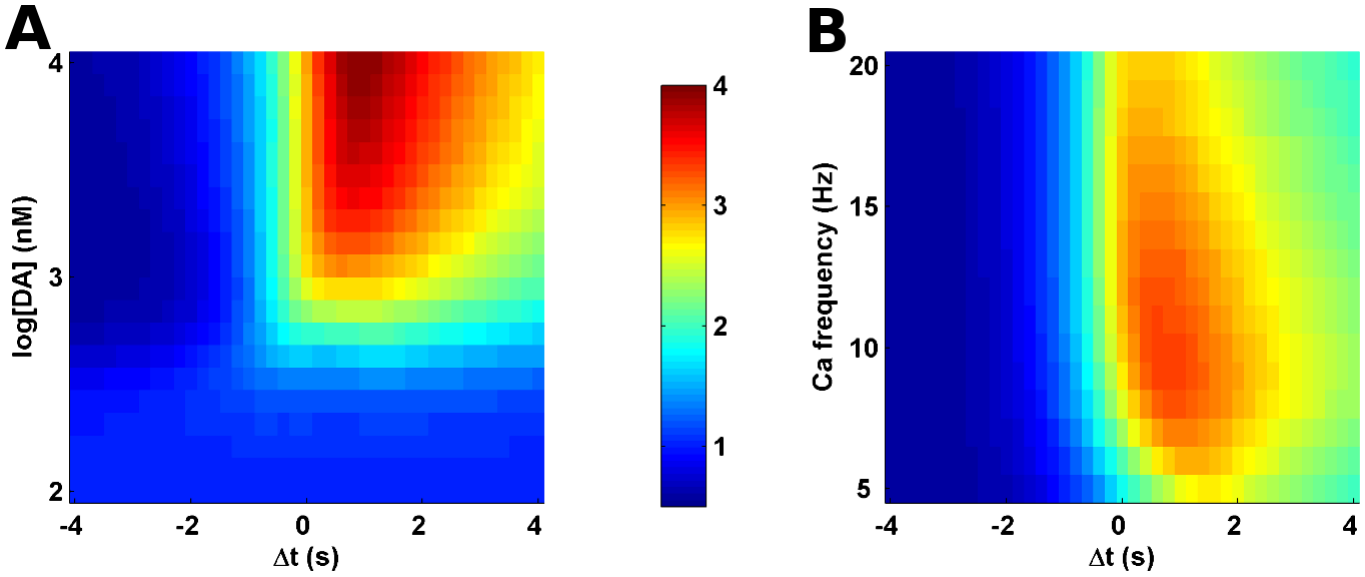
Robustness of input-interval and input-order constraints towards input signal strength. (A) The normalized area under the curve of pSubstrate response as a function of Δt and the amplitude of the dopamine (DA) transient input. The y-axis represents the log of dopamine amplitude in nanomolarity. (B) The area under the curve of pSubstrate response as a function of Δt and the calcium frequency. The calcium input consists of 10 pulses at various specified frequencies. Colors correspond to the color bar shown between the two plots. The color scale represents normalized area under the curve of pSubstrate response (pSubstrate area) in the simulations for a given condition. The values are normalized to the pSubstrate area for calcium input alone.

Amplitude of the striatal dopamine represents the reward prediction error and it could vary depending on the difference between the expected and the acquired reward [62]. In a similar fashion, the cortical activity depends on the state of the organism [63] which in turn may affect the striatal calcium signal. If the temporal constraints of the input signal integration are of generic nature then they might be relatively robust to such input variability. Thus, we looked at the effects of input variability on the calcium-dopamine integration time window. In our model the strength of effective dopamine input is represented by its amplitude whereas the effective amplitude of the calcium input is controlled by its frequency (refer “Materials and Methods”). Our simulations with varying amplitude of dopamine input indicate that there is no significant downstream response for low dopamine amplitudes, Fig 5A, as these dopamine levels are suggested to be not sufficient for significant PKA activation and DARPP-32 phosphorylation [28]. As the dopamine amplitude increases, the downstream response also increases. However, the relation between Δt and the downstream response is preserved for those cases which produced a response, Fig 5A. Similarly, the temporal constraints are preserved against the variation in the calcium input frequency as well, Fig 5B. For higher calcium frequencies, causing increased calcium levels, the input-interval constraint appears to be slightly more stringent as the range of Δt which could produce the downstream response is relatively narrower. However, in both the cases the input-order constraint of the integration process remains robust despite the changes in input strength. This relative robustness of the temporal constraints against variations in input signal properties suggests that these integration rules could be generic and not specific to certain input-signal parameters.

### Model Sensitivity Due to Perturbation in Parameters

**Fig 6:**
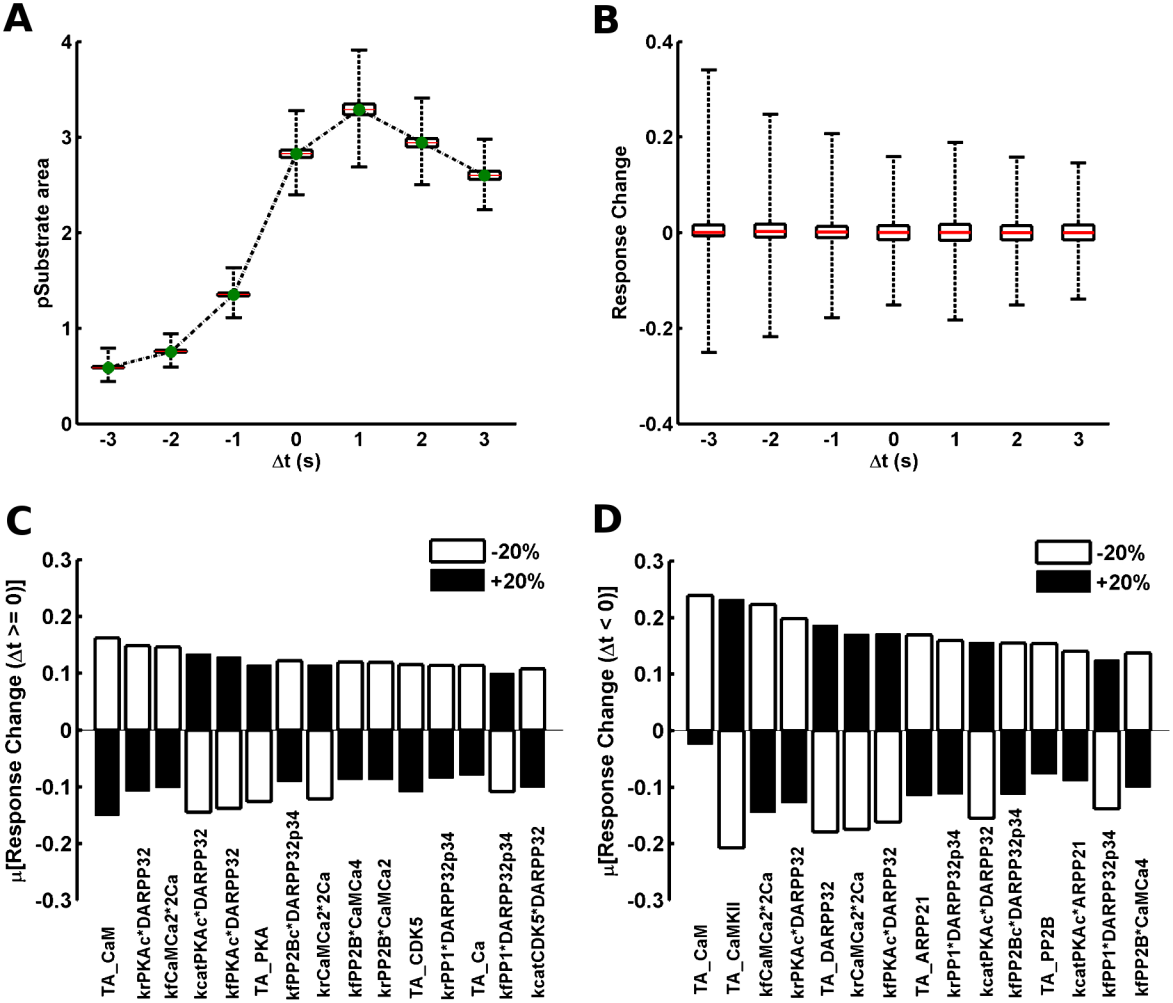
Response sensitivity towards parameter perturbation. (A) The distribution of substrate phosphorylation produced by one-at-a-time parameter perturbation (±20%) at different Δt values. At each Δt the black horizontal lines correspond to the 25^th^ and 75^th^ quartile, the red horizontal lines represent the median of the distribution and the whiskers show the spread of maximum and minimum values in the distribution. The green dots connected by dashed line are the substrate response of the base model. The pSubstrate area is normalized to without dopamine case as for previous figures. (B) The distribution of fractional (relative) change in the substrate response (pSubstrate area) produced by parameter perturbations at different Δt values. At each Δt the black horizontal lines correspond to the 25^th^ and 75^th^ quartile, the red horizontal lines represent the median of the distribution and the whiskers show the spread of maximum and minimum values in the distribution. (C) Top fifteen sensitive parameters for the positive Δt region. Y-axis represents the fractional response change due to each respective parameter averaged over all different positive Δt values (Δt ≥ 0). Empty bars correspond to the effect of negative parameter perturbation (-20%) whereas filled bars correspond to the effect of positive parameter perturbation (+20%). The following convention has been used for the name of the parameters; TA_X = total amount of X chemical species; kcatA1*A2 = catalytic rate (k_cat_) of the enzyme A1 on substrate A2; kfA1*A2 = association rate constant (k_f_) between A1 and A2; krA1*A2 = dissociation rate constant (k_r_) between A1 and A2. (D) Top fifteen sensitive parameters for the negative Δt region. Y-axis represents the fractional response change due to each respective parameter averaged over all different negative Δt values (Δt < 0).

The quantitative model built in this study is quite detailed and contains several parameters. Many of the parameters which might be important are not directly measured for the system of interest. They are rather optimized to fit experimental observations/phenotypes which emerge from the interactions of multiple parameters. However, it is important that the biological phenomena and predictions in the study should be robust with respect to changes in parameters around the selected point in the parameter space. Here, the parameters of interest are the kinetic rate constants of individual reactions and the starting amount of various conserved species. As mentioned above, the model output appears to be quite robust with respect to different parameter perturbation, Fig 2E. However, there could be certain parameters to which the output might be particularly sensitive. To identify these top sensitive parameters we perturbed all parameters one-at-a-time by ±20% of their original value. We then looked at the change in the model output produced by each of these perturbations. Fig 6A shows the distribution of substrate phosphorylation for all perturbations at different Δt values. Most of the perturbations produced a fractional response change in the range of [-0.01, +0.01] (between -1% and +1%) for all Δt, Fig 6B. However, there were some perturbations which produced higher fractional response changes, Fig 6B. Since the signaling branches responsible for the model behavior in the positive and negative Δt regions are different, the sensitive parameters for these two Δt regions could also be different. Thus, we identified the sensitive parameters separately for the two Δt regions. The response change due to each parameter averaged separately for positive (μ[response change (Δt ≥ 0)]) and negative (μ[response change (Δt < 0)]) values of Δt have been used to identify the effect of individual parameters in the respective Δt regions. The top fifteen sensitive parameters affecting the positive and negative Δt regions are shown in Fig 6C and 6D, respectively, along with the fractional response change produced due their perturbation (both +20% and -20%).

The top sensitive-parameters list for the positive Δt region includes several DARPP-32 related parameters, Fig 6C. Specifically, the kinetic parameters governing the DARPP-32 phosphorylation at Thr-34 by PKA, dephosphorylation of DARPP-32 Thr-34 by PP2B and the rate constant for the DARPP-32 mediated PP1 inhibition. The emergence of these parameters as sensitive aligns with the aforementioned critical requirement of fast DARPP-32 signaling. This could also suggest that the onset and decay kinetics of DARPP-32 phosphorylation along with the kinetics of DARPP-32 mediated PP1 inhibition is carefully regulated by these neurons. However, total amount of DARPP-32 does not significantly affect the output for positive Δt. This is because MSNs are known to express a significantly high amount (~50μM) of DARPP-32. Therefore, the total amount of DARPP-32 is not a limiting factor. Apart from these DARPP-32 related parameters the sensitive parameters for the positive Δt also included kinetic parameters for the activation of PP2B by calmodulin (CaM). Since, PP2B is responsible for the dephosphorylation of DARPP-32 at Thr-34 these PP2B activation parameters indirectly affect the state of DARPP-32 phosphorylation. This is true for the total amount of PKA and CDK5 also which are involved in the distribution of the phosphorylated states of DARPP-32.

In the case of negative Δt, the top sensitive parameters include the total amount of ARPP-21 and the kinetic parameter for ARPP-21 phosphorylation by PKA, Fig 6D. This aligns with the proposed ARPP-21 mediated effect on the negative Δt region according to our simulation results. The negative Δt also appears to be sensitive to DARPP-32 related parameters. A perturbation in total amount of DARPP-32 has an effect on the negative Δt region. This is because the estimated amount of DARPP-32 (50μM) is significantly higher than the total amount of ARPP-21 (20μM) in MSNs and both of these proteins are phosphorylated by PKA. The active PKA is a limited resource shared by DARPP-32 and ARPP-21 thus a change in the total amount DARPP-32 may affect the ARPP-21 mediated signaling.

Additionally, the total amounts of CaMKII and PP2B also appear in the list of sensitive parameters in this case. This becomes clear by considering that both CaMKII and PP2B are also the downstream targets of calmodulin similar to ARPP-21 and changes in their total amounts could affect the level of binding between phospho-ARPP-21 and Ca^2+^/calmodulin. It is also interesting to note that the total amount of calmodulin appears to be a sensitive parameter for both positive and negative Δt region. This could be because calmodulin has several interactions with the DARPP-32, ARPP-21 and CaMKII mediated signaling. The combined effects of calmodulin on the DARPP-32 and CaMKII significantly affect the gating potential of DARPP-32 signaling. Moreover, the core ARPP-21 mediated signaling relies on the calmodulin sequestration/competition thus adding further to the sensitivity of the model/predictions towards calmodulin.

## Discussion

**Fig 7:**
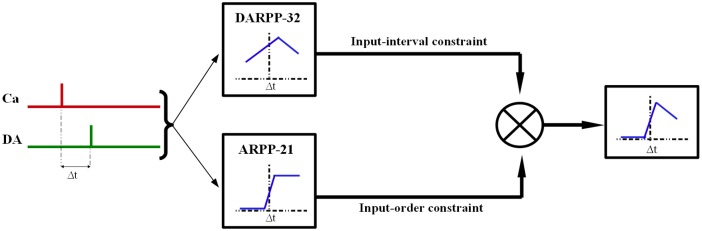
Role of DARPP-32 and ARPP-21 in the integration of calcium and dopamine transients in MSNs. DARPP-32 implements a Δt-dependent gating function for CaMKII and PP1 dependent downstream processes whereas ARPP-21 implements a Δt-dependent threshold-like function. The product of these parallel functions implemented by both phosphoproteins approximately characterizes the Δt dependence of the final downstream response. The blue curves within the boxes schematically represent the Δt-dependence of the response implemented at each step.

The subcellular integration of the NMDA-dependent calcium and dopamine by D1R-expressing MSNs is believed to be an important biological process for reward learning. In this kinetic modeling study, we propose a novel mechanism which could explain the emergence of the observed input-interval and input-order constraints on the integration of calcium and dopamine. These temporal constraints could emerge as a result of the coordinated signaling through two striatally enriched phosphoproteins, namely DARPP-32 and ARPP-21, Fig 7. Both of these phosphoproteins are localized significantly in the dendritic compartments of MSNs and this co-localization could indicate a possible functional cooperation [64]. The major hypotheses, observations and testable-predictions made by this study are listed in Table 2.

**Table 2:** Summary of the hypotheses/predictions/observations highlighted by this modeling study.

**Proposed hypotheses**			
1. DARPP-32 imposes the requirement for the temporal proximity of the calcium and the dopamine inputs. It mediates the dopamine dependent gating of the CaMKII signaling by transiently inhibiting PP1 which is otherwise sufficiently strong to counteract the CaMKII response to calcium alone.			
2. ARPP-21 imposes the requirement that dopamine input should follow the calcium input due to its property to sequester Ca^2+^/calmodulin (required for CaMKII activation) if dopamine precedes calcium.			
3. The important aspects of the temporal constraint on the calcium-dopamine integration emerge from the coordinated activity of DARPP-32 and ARPP-21 where DARPP-32 is largely responsible for the input-interval constraint and ARPP-21 is responsible for the input-order constraint.			
4. The effect of ARPP-21 spills-over to a subsequent trial in a multi-trial scenario in an inter-trial interval dependent manner.			
**Testable Predictions**			
**Stimulus**	**Condition**	**Marker**	**Prediction/Observation**
Transient (calcium + dopamine) inputs	Wild-type	CaMKII/PP1 substrate phosphorylation	The response is strong only if the dopamine input (reinforcement) follows the calcium input. If the dopamine transient precedes the calcium then there is very low response. For a certain positive interval (dopamine follows calcium) between the calcium and the dopamine inputs the response reaches maximum and then as the interval increases the response starts to decline.
		CaMKII activation	The amplitude of CaMKII activation is high only if dopamine follows the calcium input. If dopamine precedes calcium then the CaMKII amplitude is significantly low.
	DARPP-32 mutant at Thr-34	CaMKII/PP1 substrate phosphorylation	No significant response for any order and interval between calcium and dopamine inputs.
		CaMKII activation	The amplitude of CaMKII activation is high only if dopamine follows the calcium input. If dopamine precedes calcium then the CaMKII amplitude is significantly low. This is similar to the wild-type case except that the duration of CaMKII activation is shorter due to active PP1.
	ARPP-21 mutant at Ser-55	CaMKII/PP1 substrate phosphorylation	The response is strong irrespective of the order of calcium and dopamine inputs unlike the wild type scenario where response is only elicited when dopamine follows calcium. As the interval between the two inputs increase the response starts to decline.
		CaMKII activation	The amplitude of CaMKII activation is always high irrespective of the order of calcium and dopamine inputs. The amplitude is even not affected by the presence or absence of dopamine.
	Calmodulin overexpression	CaMKII/PP1 substrate phosphorylation	No significant effect of dopamine or the order of the two inputs.
		CaMKII activation	No significant effect of dopamine or the order of the two inputs.
Two instances of transient (calcium + dopamine) inputs separated by varying inter-trial interval (ITI).	Wild-type	CaMKII/PP1 substrate phosphorylation	For smaller ITI values, the response is significantly lower for the second set of (calcium + dopamine) input compared to the response for first set of inputs. For higher ITI values the response for both sets of inputs are similar.
	ARPP-21 mutant at Ser-55	CaMKII/PP1 substrate phosphorylation	No significant effect of the first trial on the response of the second trial.

In our simulations, dopamine acts as a gate for the calcium-triggered CaMKII/PP1 signaling and this aligns with known observations [8,9,17]. This gating is mediated by the phosphorylation of DARPP-32 in response to dopamine-triggered cAMP/PKA signaling. DARPP-32 mainly acts by inhibiting PP1. If PP1 is not inhibited then it is sufficiently strong to counteract the effect of CaMKII. Thus, the coincidence of a calcium-triggered CaMKII activation and dopamine-dependent PP1 inhibition leads to a significant substrate phosphorylation. Such cAMP/PKA dependent effect on CaMKII signaling is not just specific to striatum. A similar phenomenon has been observed in the hippocampus as well, where it is mediated by Inhibitor-1, a homologue of DARPP-32 [65,66].

The transient nature of both CaMKII activation and PP1 inhibition limits the possible time-interval (Δt) between the respective calcium and dopamine inputs, which could be synergistically integrated. Moreover, the PP1 inhibition kinetics should be fast enough to match the temporal characteristics of CaMKII activation. In other words, the PP1 inhibition should occur before the transient CaMKII activation fades away if these two were to interact. This requires particularly fast kinetics for the cAMP/PKA signaling axis which is already known to be the case in striatal neurons [40]. Recent observations in the dendritic compartments of these neurons indicate that DARPP-32 mediated effect is manifested within a few seconds, thus supporting the requirement for fast DARPP-32-dependent PP1 inhibition [9].

Even though the transient DARPP-32-dependent effect could implement the observed input-interval constraint, according to our results, DARPP-32 signaling alone may not distinguish between the order of calcium and dopamine incidence. Moreover, the molecular identity of the substance which could make the striatal CaMKII/PP1 signaling sensitive to the temporal order of calcium and dopamine is not clear. However, as presented above, ARPP-21 has the potential to implement an input-order dependent filtering mechanism by acting as a competitor to CaMKII. This could enable the striatal signaling machinery to distinguish between the incidence order of calcium and dopamine. When ARPP-21 is phosphorylated by a preceding dopamine it turns into a potent sequestering agent for subsequent calcium-triggered Ca^2+^/calmodulin, thus reducing the available Ca^2+^/calmodulin for CaMKII activation. Similar to the DARPP-32 mediated phenomenon, this ARPP-21 mediated effect also demands fast phosphorylation kinetics.

As aforementioned, the ARPP-21 mediated mechanism rests on the idea that ARPP-21, upon phosphorylation, competes with CaMKII for the available Ca^2+^/calmodulin. This competition stipulates that the amount of calmodulin be lower than the collective amount of its possible binding partners, i.e. Ca^2+^/calmodulin is a limiting resource. If Ca^2+^/calmodulin is not a limited resource then pARPP-21 and CaMKII do not have to compete with each other, thereby reducing the inhibitory effect of ARPP-21 on CaMKII activation. This in turn will eliminate the input-order sensitivity of the calcium-dopamine integration. Even though the absolute amount of calmodulin could be high in many cell types, various studies suggest that free calmodulin is indeed a limiting resource [67–69]. The same has been inferred for neurons as well [70]. Moreover, such a limit on available Ca^2+^/calmodulin seems to exist also in MSNs [15] thus supporting this necessary requirement for the proposed mechanism.

In addition to CaMKII, Ca^2+^/calmodulin is also responsible for the activation of PP2B which dephosphorylates DARPP-32 Thr34. However, unlike CaMKII, pARPP-21 does not have any significant effect on the level of PP2B activation, for transient input signals, in our signaling model because the affinity of Ca^2+^/calmodulin towards PP2B is assumed to be very high (K_d_ in subnanomolar range; [71]) compared to the affinity of Ca^2+^/calmodulin towards pARPP-21 (K_d_ in tens of nanomolar). Thus, the current study does not predict any significant ARPP-21 mediated effect on DARPP-32 dephosphorylation within the assumed parameter space for transient input signals. However, this does not rule out such a regulation. Rather this could be due to the insufficiency of data to tune this aspect of the current signaling model.

Even though the role of striatal ARPP-21 is not clear, it seems to be involved in addiction and motivational behavior [54,72]. The current study predicts a clear physiological role for ARPP-21 in the context of reward learning. We also predict that the downregulation of ARPP-21 could significantly hinder the ability of MSNs to identify the temporal order of calcium and dopamine signals. On a behavioral level, this implies an increased appearance of association between a stimulus and a reinforcement which preceded the stimulus. This kind of backward association tends to eliminate the predictability of a conditioned stimulus. Thus, we also predict that an ARPP-21 downregulated phenotype could have an aberrant causality perception, more specifically deficits in associating a reward with the optimal cue in a complex environment.

Apart from imposing the input-order constraint on the calcium-dopamine integration, our simulations also suggest that ARPP-21 could impose an inter-trial refractoriness as a function of the ITI in a multi-trial conditioning scenario. This could imply that an MSN, or its dendritic segment, which is potentiated in the recent past would resist a further potentiation within a refractory period. This could mean that multiple trials with shorter ITIs may not be completely independent to each other. As the ITI increases the trials start to become decoupled. Such, an inverse relation between conditioned response and ITI is well known for classical conditioning [73,74]. Although there may be several factors responsible for such a relation, ARPP-21 could be one of the molecular players involved in this.

This study highlights the possible role of phosphoproteins in controlling the temporal aspects of striatal signal transduction. Furthermore, we also attempt to illustrate the idea that different aspects of a single phenomenon (temporal constraint in this case) could be distributed among parallel signaling modules (DARPP-32 and ARPP-21) and do not necessarily have to be implemented by a single signaling node. One such single-signaling node which is believed to be responsible for similar temporal constraints in other systems, like Aplysia serotonin response, is Ca^2+^/calmodulin stimulated Adenylyl cyclase, which exhibits calcium-dependent priming on the G-protein activation [75,76]. A similar Ca^2+^/calmodulin stimulated Adenylyl cyclase, Adenylyl cyclase type I (AC1), has also been suggested to be involved in the emergence of striatal input-interval and input-order constraints [9]. AC1 is known to be synergistically activated by G_s_ (G_olf_) G-protein and Ca^2+^/calmodulin and is known to act as a coincidence detector for these two signals [77]. Even though the activation pattern of AC1 is not clear for transient inputs, there could exist kinetic parameter sets for which AC1 may enforce the temporal constraints (see Fig A a,b in S1 Text). However, the expression of AC1 is low in the striatum of adult organisms even though it may exist in the early stages of development [78,79]. During postnatal development most of the AC1 is replaced by AC5, and AC5 is the predominant functional enzyme in adult organisms [31,79,80]. AC5, which we use in our model, is strongly activated by the G_olf_ and does not display any calcium-dependent synergy [81]. It could be a possible scenario that lower amounts of AC1 may still be expressed in adult MSNs making the overall AC population a mixture of AC5 and AC1. In such a mixed AC population, which contains both AC5 and AC1, the overall behavior may be dominated by the AC with the higher fraction (see Fig A c in S1 Text). For example, the AC1 dependent temporal constraints may emerge, irrespective of ARPP-21, if the fraction of AC1 is high in the total AC (see Fig A c in S1 Text). However, for a scenario consistent with striatal data, i.e. high AC5 and low AC1 the contribution of AC1 is overshadowed by the bulk response of AC5 and the overall response appears to be similar to the system containing only AC5 (see Fig A c in S1 Text). Thus, in striatum, the bulk AC5 dependent cAMP response may significantly dampen any AC1 dependent component of temporal constraints due to the high amount of AC5.

On the other hand, the DARPP-32/ARPP-12 dependent mechanism proposed here is quite generalizable to various systems consisting of different AC isoforms. Even though we focused on the potential role of DARPP-32 and ARPP-21, they might just be part of a much wider signaling mechanism to produce the temporal constraints on striatal calcium-dopamine integration. For example, the activation of other signaling processes, like PKA, have also been observed to display the input-interval and input-order constraints [9]. Thus, further exploration of the wider signaling network involved in this process is of interest. For example, it is known that several other crosstalk points exist between calcium and dopamine signaling axis at various downstream levels of the postsynaptic signaling [50,82–84]. Some of them might play a role in further fine-tuning of the temporal constraints.

It is interesting to note that there are brain regions other than the striatum, e.g. the amygdaloid complex and the bed nucleus of the stria terminalis (BNST), where the co-expression of DARPP-32 and ARPP-21 is known to exist [64,85–87]. Both amygdala and BNST are critical for fear conditioning and they play a role in the association between cue/context to a stress-inducing unconditioned response [88]. Thus, the combined DARPP-32/ARPP-21 signaling may play similar roles to the ones discussed in this study for these brain regions. Thus, the phosphoprotein-dependent mechanism proposed in this study could represent a more general brain-wide signaling motif responsible for the implementation of temporal constraints on subcellular signal integration of environmental cues and reinforcement.

## Supporting Information

S1 Text Supporting information about the model parameters and the exploration of additional signaling regulation. (PDF)
