# Supplementary material for: Role of DARPP-32 and ARPP-21 in the Emergence of Temporal Constraints on Striatal Calcium and Dopamine Integration
Source: PLoS Comput Biol. 2016 Sep 1;12(9):e1005080. doi: 10.1371/journal.pcbi.1005080 (PMC5008828; doi:10.1371/journal.pcbi.1005080)
Supplement: S1 Text — (PDF) [file pcbi.1005080.s001.pdf]

S1 Text

Reactions and parameters used in the model

Following are the lists of all reactions and the respective reaction rate parameters of the modeled signaling network. The rate parameters and total amounts were taken from the literature whenever possible, but in most cases they resulted from the manual fitting of the model to phenotypic data. The chemical species "totalpCaMKIIpsd" represents the total amount of all calmodulin bound and phosphorylated CaMKII species in the PSD domain (with the suffix –psd).

Table A: Reversible reactions

**Reactions**	**Forward Reaction rates (1/(nM*s))**	**Reverse Reaction rates (1/s)**
D1RGolf + DA ↔ D1RDAGolf	0.05	250
D1R + DA ↔ D1RDA	0.05	250
D1R + Golf ↔ D1RGolf	0.06	250
Golf + D1RDA ↔ D1RDAGolf	0.06	250

AC5 + GaolfGTP ↔ AC5GaolfGTP	10	1
AC5GaolfGTP + ATP ↔ AC5GaolfGTP*ATP	0.00255	1
AC5 + Ca ↔ AC5Ca	0.001	10
AC5Ca + GaolfGTP ↔ AC5CaGaolfGTP	10	1
AC5 + ATP ↔ AC5*ATP	0.0001	1
AC5Ca + ATP ↔ AC5Ca*ATP	7.50E-05	1
AC5CaGaolfGTP + ATP ↔ AC5CaGaolfGTP*ATP	0.0013	1
GaolfGTP + AC5*ATP ↔ AC5GaolfGTP*ATP	10	1
GaolfGTP + AC5Ca*ATP ↔ AC5CaGaolfGTP*ATP	10	1
Ca + AC5*ATP ↔ AC5Ca*ATP	0.001	10

cAMP + PDE4 ↔ PDE4*cAMP	0.03	1
PDE10 + 2cAMP ↔ PDE10c	1.00E-06 (1/(nM^2*s))	1
cAMP + PDE10 ↔ PDE10*cAMP	0.1	2
cAMP + PDE10c ↔ PDE10c*cAMP	0.1	2

cAMP + PKA ↔ PKAcAMP2	0.026	350
cAMP + PKAcAMP2 ↔ PKAcAMP4	0.0346	50
PKAcAMP4 ↔ PKAc + PKAreg	50	0.03

CaM + Ca ↔ CaMCa2	0.006	20
CaMCa2 + Ca ↔ CaMCa4	0.1	1000

PP2B + CaM ↔ PP2BCaM	0.1	3000
CaMCa4 + PP2B ↔ PP2Bc	0.1	0.003
Ca + PP2BCaM ↔ PP2BCaMCa2	0.006	0.0002
Ca + PP2BCaMCa2 ↔ PP2Bc	0.1	100
CaMCa2 + PP2B ↔ PP2BCaMCa2	0.1	0.03

PKAc + DARPP32 ↔ PKAc*D32	0.01	200
PKAc + B56PP2A ↔ PKAc*B56PP2A	0.001	0.3
D32p34 + PP1 ↔ PP1D32p34	1	1.5
CDK5 + DARPP32 ↔ CDK5*D32	0.001	100
D32p75 + PKAc ↔ PKAcD32p75	0.1	100
B72PP2A + Ca ↔ B72PPA2Ca	0.01	10
B56PP2Ap + D32p75 ↔ B56PP2Ap*D32p75	0.015	100
B72PP2A + D32p75 ↔ B72PP2A*D32p75	0.008	100
D32p75 + B72PPA2Ca ↔ B72PP2ACa*D32p75	0.015	100
D32p34 + PP2Bc ↔ PP2Bc*D32p34	1.3	0.1
D32p75 + B56PP2A ↔ B56PP2A*D32p75	0.008	100
D32p34 + B72PPA2Ca ↔ B72PP2ACa*D32p34	0.0005	1
D32p34 + B72PP2A ↔ B72PP2A*D32p34	0.0005	1

ARPP21 + PKAc ↔ PKAc*ARPP21	0.045	200
pARPP21 + CaMCa4 ↔ CaMCa4pARPP21	0.5	10
pARPP21 + B72PPA2Ca ↔ B72PPA2Ca*pARPP21	0.007	100
pARPP21 + B72PP2A ↔ B72PP2A*pARPP21	0.004	100
pARPP21 + B56PP2Ap ↔ B56PP2Ap*pARPP21	0.007	100
pARPP21 + B56PP2A ↔ B56PP2A*pARPP21	0.004	100

CaMKII + CaMCa4 ↔ CaMKIIc	0.1	40
CaMCa2 + CaMKII ↔ CaMKIICaMCa2	0.1	400
CaM + CaMKII ↔ CaMKIICaM	0.1	4000
CaMKIICaMCa2 + Ca ↔ CaMKIIc	0.1	100
Ca + CaMKIICaM ↔ CaMKIICaMCa2	0.006	2

pCaMKIICaMCa2 + Ca ↔ pCaMKIIc	0.1	10
pCaMKIICaM + Ca ↔ pCaMKIICaMCa2	0.006	2
pCaMKII + CaMCa4 ↔ pCaMKIIc	0.1	0.4
pCaMKII + CaMCa2 ↔ pCaMKIICaMCa2	0.1	40
pCaMKII + CaM ↔ pCaMKIICaM	0.1	400

pCaMKIIc ↔ pCaMKIIcpsd	0.5	0.001
pCaMKIICaMCa2 ↔ pCaMKIICaMCa2psd	0.5	0.001
pCaMKIICaM ↔ pCaMKIICaMpsd	0.5	0.001
pCaMKII ↔ pCaMKIIpsd	0.5	0.001
CaMKIICaM ↔ CaMKIICaMpsd	0.5	0.5
CaMKIICaMCa2 ↔ CaMKIICaMCa2psd	0.5	0.5
CaMKIIc ↔ CaMKIIcpsd	0.5	0.5

CaMCa4 + pCaMKIIpsd ↔ pCaMKIIcpsd	0.1	0.4
pCaMKIIpsd + CaMCa2 ↔ pCaMKIICaMCa2psd	0.1	40
CaM + pCaMKIIpsd ↔ pCaMKIICaMpsd	0.1	400
pCaMKIICaMpsd + Ca ↔ pCaMKIICaMCa2psd	0.006	2
pCaMKIICaMCa2psd + Ca ↔ pCaMKIIcpsd	0.1	10
CaMCa4 + CaMKIIpsd ↔ CaMKIIcpsd	0.1	40
CaMCa2 + CaMKIIpsd ↔ CaMKIICaMCa2psd	0.1	400
CaM + CaMKIIpsd ↔ CaMKIICaMpsd	0.1	4000
CaMKIICaMCa2psd + Ca ↔ CaMKIIcpsd	0.1	100
Ca + CaMKIICaMpsd ↔ CaMKIICaMCa2psd	0.006	2
pCaMKIIpsd + PP1 ↔ PP1*pCaMKIIpsd	0.0008	1

Substrate + totalActCaMKIIpsd ↔ totalpCaMKIIpsd*Substrate	0.0005	10
pSubstrate + PP1 ↔ PP1*pSubstrate	0.0005	1

Table B: Irreversible reactions

**Reactions**	**Reaction rates (1/s)**
D1RDAGolf → Gbgolf + D1RDA + GaolfGTP	15
GaolfGTP → GaolfGDP	30
GaolfGDP + Gbgolf → Golf	100

AC5GaolfGTP*ATP → cAMP + AC5GaolfGTP	50

cAMP + AC5GaolfGTP → AC5GaolfGTP*ATP	2.55
AC5*ATP → cAMP + AC5	1
cAMP + AC5 → AC5*ATP	0.002
AC5Ca*ATP → cAMP + AC5Ca	0.5
cAMP + AC5Ca → AC5Ca*ATP	0.00075
AC5CaGaolfGTP*ATP → cAMP + AC5CaGaolfGTP	25
cAMP + AC5CaGaolfGTP → AC5CaGaolfGTP*ATP	0.65
AC5GaolfGTP → AC5 + GaolfGDP	1
AC5CaGaolfGTP → AC5Ca + GaolfGDP	1
AC5GaolfGTP*ATP → AC5*ATP + GaolfGDP	1
AC5CaGaolfGTP*ATP → AC5Ca*ATP + GaolfGDP	1

PDE4*cAMP → PDE4 + AMP	2.5
PDE10*cAMP → PDE10 + AMP	3
PDE10c*cAMP → PDE10c + AMP	10

PKAc*D32 → D32p34 + PKAc	10
PKAc*B56PP2A → B56PP2Ap + PKAc	0.2
CDK5*D32 → CDK5 + D32p75	10
B56PP2Ap*D32p75 → B56PP2Ap + DARPP32	8
B72PP2A*D32p75 → B72PP2A + DARPP32	1.5
B72PP2ACa*D32p75 → B72PPA2Ca + DARPP32	8
B56PP2A*D32p75 → B56PP2A + DARPP32	1.5
B72PP2ACa*D32p34 → B72PPA2Ca + DARPP32	3
B72PP2A*D32p34 → DARPP32 + B72PP2A	3
PP2Bc*D32p34 → DARPP32 + PP2Bc	1.2
B56PP2Ap → B56PP2A	0.008

PKAc*ARPP21 → PKAc + pARPP21	10
B72PPA2Ca*pARPP21 → ARPP21 + B72PPA2Ca	10
B72PP2A*pARPP21 → ARPP21 + B72PP2A	1
B56PP2Ap*pARPP21 → ARPP21 + B56PP2Ap	10
B56PP2A*pARPP21 → ARPP21 + B56PP2A	1

CaMKIIpsd → CaMKII	0.5
CaMKIIcpsd → pCaMKIIcpsd	According to the autophosphorylation fitting discussed in [24] with $10 s^{-1}$ as maximum rate.
pCaMKII → CaMKII	0.1

CaMKIIc → pCaMKIIc	According to the autophosphorylation fitting discussed in [24] with $10 s^{-1}$ as maximum rate.
PP1*pCaMKIIpsd → PP1 + CaMKIIpsd	1

totalpCaMKIIpsd*Substrate → pSubstrate + totalActCaMKIIpsd	10
PP1*pSubstrate → PP1 + Substrate	10

Table C: Initial amounts of different species

**Species name**	**Initial Amount (nM)**
DARPP32	50000
CaM	9000
CaMKII	20000
PP1	3000
PP2B	4000
B72PP2A	2000
PKA	1200
PDE4	2000
PDE10	700
CDK5	1800
AC5	700
Ca	60
DA	20
B56PP2A	2000
ATP	5000000
D1R	2000
Golf	2000
ARPP21	20000
Substrate	3000

Exploration of AC1 contribution

AC5 is the Adenylyl cyclase isoform used in our signaling model as it is believed to be the predominantly functional AC isoform in striatum [31]. However, it has been previously indicated that AC1 could also play a role in enforcing the temporal constraints on striatal calcium-dopamine integration [9]. We quantitatively tested whether the coincidence detection by AC1 is sufficient to explain the input-interval and input-order constraints. Our simulations indicated that there could exist a parameter space (reaction parameters in Table D) in which AC1 alone could enforce some temporal constraints both on PKA and the downstream CaMKII/PP1 substrate phosphorylation, (Fig A a,b). Even though the expression of AC1 is low in the striatum of adult organisms it may constitute some fraction of the total striatal AC. Thus, we looked at the relative contribution of AC1 in the striatal temporal constraints. We tested this by exploring whether the presence of AC1 in the total AC population is sufficient to enforce the input-interval and input-order constraints. According to a scan with varying amounts of AC1 (0 to 100%) in the striatal AC population, the effect of AC5 dominates unless the amount of AC1 is significantly higher than the AC5, Fig A c (upper panel). Thus, in such a mixed AC population, which contains both AC5 and AC1, the overall behavior may be dominated by the AC with the higher fraction. Since, AC5 is the dominant fraction in striatum the overall behavior may closely follow the AC5 behavior, i.e. AC alone may not be sufficient enough to enforce the input-order constraint. In such a scenario, ARPP-21 appeared to be important for enforcing the input-order constraints aligning with the results from the original model, Fig A c (lower panel). A cross-section of the scans (with and without ARPP-21) with 20% of AC1 and 80% of AC5, i.e. high AC5 and low AC1 similar to striatal conditions, (Fig A c, upper and lower traces) is very similar to the response produced by the original signaling model in the main text (Fig 1G, 2C), thereby suggesting no significant effect of AC1 in the parameter space of the current model. However, this does not rule out the possibility about the role of AC1 in a scenario where different AC isoforms are enriched in different compartments (e.g. high AC1 in outer dendrites and high AC5 in soma and proximal dendrites).

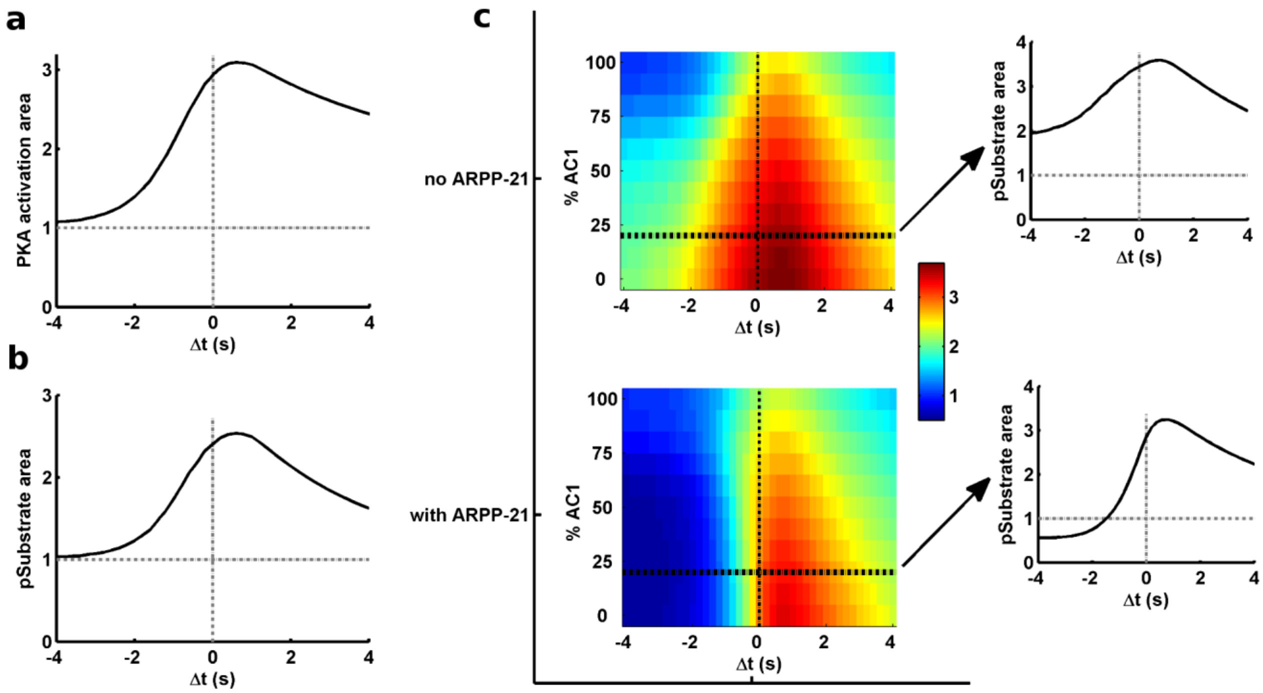

Fig A. *Effect of AC1 in the model. (a) PKA activation produced by the model containing AC1 (refer Table D for AC1 reaction parameters) instead of AC5 as a function of Δt. The responses are normalized to the response in the case with calcium alone. (b) Downstream phosphorylated CaMKII/PP1 substrate (pSubstrate area) produced by the model containing AC1 instead of AC5 as a function of Δt. The responses are normalized to the response in the case with calcium alone. (c) Downstream CaMKII/PP1 substrate phosphorylation (pSubstrate area) produced by model which includes both AC5 and AC1 as a function of Δt and percentage of AC1 in the total AC population. Upper panel is for the signaling network without ARPP-21 and the lower panel is for the model containing ARPP-21. Colors in the colorbar represent the substrate phosphorylation (pSubstrate area). The right traces correspond to the cross-section of substrate response for the black dashed line (20% AC1 and 80% AC5) in the respective heatmaps without and with ARPP-21 cases. The responses are normalized to the response in the case with calcium alone.*

Table D: AC1 reaction parameters (used in Fig. A)

**Reversible Reactions**	**Reaction rates (1/(nM*s))**	**Reaction rates (1/s)**
CaMCa4 + AC1 ↔ AC1CaMCa4	0.005	0.1
GaolfGTP + AC1CaMCa4 ↔ AC1CaMCa4GaolfGTP	10	1

GaolfGTP + AC1 ↔ AC1GaolfGTP	10	1
AC1GaolfGTP + CaMCa4 ↔ AC1CaMCa4GaolfGTP	0.005	0.1
AC1 + ATP ↔ AC1*ATP	0.0001	1
AC1CaMCa4 + ATP ↔ AC1CaMCa4*ATP	0.001	1
AC1GaolfGTP + ATP ↔ AC1GaolfGTP*ATP	0.0003	1
AC1CaMCa4GaolfGTP + ATP ↔ AC1CaMCa4GaolfGTP*ATP	0.005	1
CaMCa4 + AC1*ATP ↔ AC1CaMCa4*ATP	0.005	0.1
GaolfGTP + AC1CaMCa4*ATP ↔ AC1CaMCa4GaolfGTP*ATP	10	1
GaolfGTP + AC1*ATP ↔ AC1GaolfGTP*ATP	10	1
AC1GaolfGTP*ATP + CaMCa4 ↔ AC1CaMCa4GaolfGTP*ATP	5.00E-03	0.1
**Irreversible Reactions**	**Reaction rates (1/s)**	
AC1GaolfGTP → GaolfGDP + AC1	1	
AC1CaMCa4GaolfGTP → AC1CaMCa4 + GaolfGDP	1	
AC1GaolfGTP*ATP → GaolfGDP + AC1*ATP	1	
AC1CaMCa4GaolfGTP*ATP → AC1CaMCa4*ATP + GaolfGDP	1	
AC1*ATP → AC1 + cAMP	1	
AC1CaMCa4*ATP → AC1CaMCa4 + cAMP	20	
AC1GaolfGTP*ATP → AC1GaolfGTP + cAMP	5	
AC1CaMCa4GaolfGTP*ATP → AC1CaMCa4GaolfGTP + cAMP	100	
cAMP + AC1 → AC1*ATP	0.002	
cAMP + AC1CaMCa4 → AC1CaMCa4*ATP	0.42	
cAMP + AC1GaolfGTP → AC1GaolfGTP*ATP	0.03	
cAMP + AC1CaMCa4GaolfGTP → AC1CaMCa4GaolfGTP*ATP	10.1	

Exploration of Ca^{2+}/CDK5/PDE4 regulation on the temporal constraints

CDK5 mediated phosphorylation of PDE4B impinges upon the basal level of cAMP in striatum [82]. Moreover, calcium elevation has been reported to the phosphorylation of CDK5 [84]. Thus, there may be a possible effect of this Ca^{2+}/CDK5/PDE4 regulation on the input-interval and input-order constraints. To explore the effect of this CDK5 dependent PDE4 regulation we included this regulation in our signaling network.

This additional regulation assumes the following:

1. CDK5 phosphorylates PDE4 [82]
2. PDE4 phosphorylation is more effective by the phosphorylated CDK5 (assumed to include the calcium-dependent regulation).
3. Calcium leads to the phosphorylation of CDK5 [84].

We constrained the parameters of this additional regulation to reproduce all the target phenotypes (Table 1 in the main text; including the basal and stimulated levels of cAMP and DARPP-32 phospho states). Fig B a1 shows the increase in CDK5 phosphorylation due to calcium signaling in this test model. This increase in the CDK5 phosphorylation results in an increase in the phosphorylation of PDE4, Fig B a2. We then used this test model to see the effect of this PDE4 regulation on the relation between the downstream response and Δt. The simulation results of this updated signaling model suggest that there is no significant effect of this additional PDE4 regulation, Fig B b (solid black line), compared to the original model, Fig B b (grey line). In the current signaling network PDE10 exerts a stronger control on the cAMP signaling than the PDE4 [40]. In order to inspect whether this efficacy of PDE10 is masking the possible effect of PDE4 regulation, we completely removed PDE10 while keeping PDE4 and CDK5/PDE4 regulation from the model and recalibrated it to the phenotypes. The response of this updated version without PDE10, Fig B b (dashed black line), is also not significantly different from the original model response. Knocking out DARPP-32 and ARPP-21 in this model with CDK5/PDE4 regulation and no PDE10 suggest that the input-interval and input-order constraints are produced by DARPP-32 and ARPP-21 signaling in this model also. Thus according to our simulations there appears to be no significant effect of Ca^{2+}/CDK5/PDE4 regulation on the temporal constraints in the current parameter space of the signaling network.

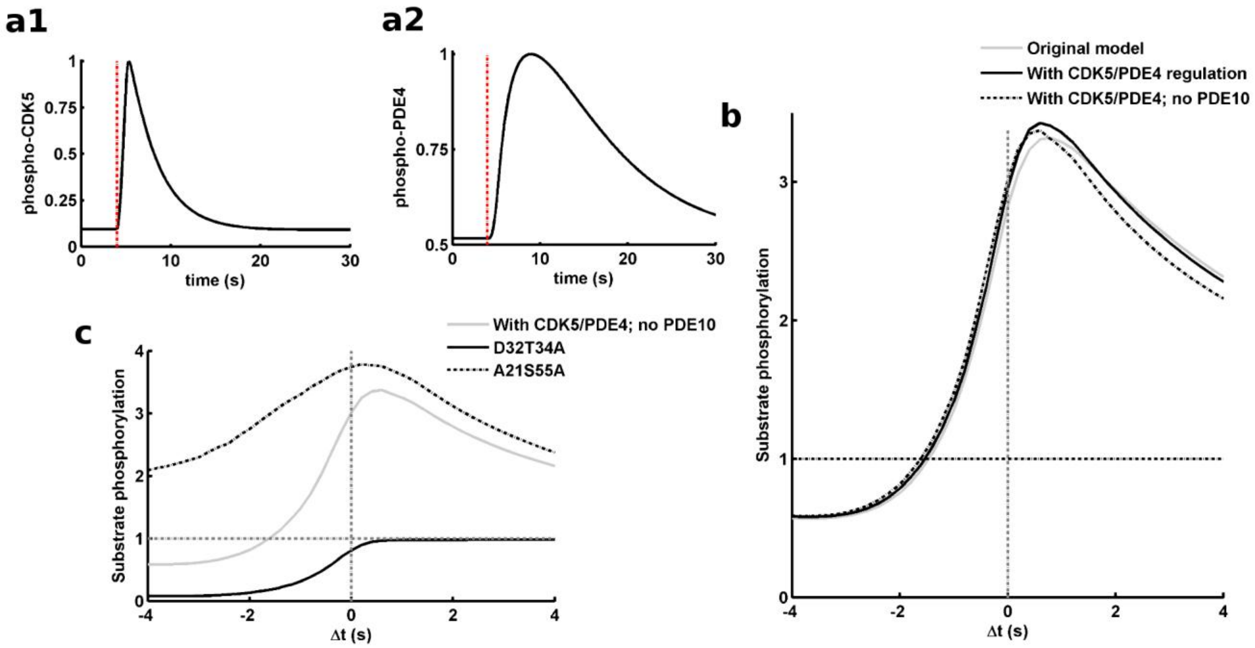

Fig B. *Scenario when model includes the Ca^{2+}/CDK5/PDE4 regulation. (a1) Phosphorylation of CDK5 in response to a calcium input. The vertical red dashed line marks the start time of calcium input. (a2) CDK5 mediated phosphorylation of PDE4 in response to a calcium input. The vertical red dashed line marks the start time of calcium input. (b) The downstream CaMKII/PP1 substrate response as a function of Δt for the original model, model with CDK5/PDE4 regulation included and model with CDK5/PDE4 regulation but without PDE10. The responses are normalized to the response in the case with calcium alone. (c) The downstream response of DARPP-32 (D32T43A) and ARPP-21 (A21S55A) mutant case for the model with CDK5/PDE4 regulation but without PDE10. The responses are normalized to the response in the case with calcium alone.*
